# Prospects for detecting early warning signals in discrete event sequence data: Application to epidemiological incidence data

**DOI:** 10.1371/journal.pcbi.1007836

**Published:** 2020-09-22

**Authors:** Emma Southall, Michael J. Tildesley, Louise Dyson

**Affiliations:** 1 EPSRC & MRC Centre for Doctoral Training in Mathematics for Real-World Systems, University of Warwick, Coventry, UK; 2 Zeeman Institute for Systems Biology and Infectious Disease Epidemiology Research, Mathematics Institute and School of Life Sciences, University of Warwick, Coventry, UK; Institute for Disease Modeling, UNITED STATES

## Abstract

Early warning signals (EWS) identify systems approaching a critical transition, where the system undergoes a sudden change in state. For example, monitoring changes in variance or autocorrelation offers a computationally inexpensive method which can be used in real-time to assess when an infectious disease transitions to elimination. EWS have a promising potential to not only be used to monitor infectious diseases, but also to inform control policies to aid disease elimination. Previously, potential EWS have been identified for prevalence data, however the prevalence of a disease is often not known directly. In this work we identify EWS for incidence data, the standard data type collected by the Centers for Disease Control and Prevention (CDC) or World Health Organization (WHO). We show, through several examples, that EWS calculated on simulated incidence time series data exhibit vastly different behaviours to those previously studied on prevalence data. In particular, the variance displays a decreasing trend on the approach to disease elimination, contrary to that expected from critical slowing down theory; this could lead to unreliable indicators of elimination when calculated on real-world data. We derive analytical predictions which can be generalised for many epidemiological systems, and we support our theory with simulated studies of disease incidence. Additionally, we explore EWS calculated on the rate of incidence over time, a property which can be extracted directly from incidence data. We find that although incidence might not exhibit typical critical slowing down properties before a critical transition, the rate of incidence does, presenting a promising new data type for the application of statistical indicators.

## Author summary

The threat posed by infectious diseases has a huge impact on our global society. It is therefore critical to monitor infectious diseases as new data becomes available during control campaigns. One obstacle in observing disease emergence or elimination is understanding what influences noise in the data and how this fluctuates when cases near to zero. The standard type of data collected is the number of new cases per day/month/year but mathematical modellers often focus on data such as the total number of infectious people, due to its analytical properties. We have developed a methodology to monitor the standard type of data to inform when a disease is approaching emergence or disease elimination. We have shown computationally how fluctuations change as timeseries data gets closer towards a tipping point and our insights highlight how these observed changes can be strikingly different when calculated on different types of data.

## Introduction

One of the greatest challenges in society today is the burden of infectious diseases, affecting public health and economic stability all over the world. Infectious diseases disproportionately affect individuals in poverty, with millions of those suffering daily from diseases that are considered eradicable. The potential for eradicating diseases such as polio, guinea worm, measles, mumps or rubella is immense (International Task Force for Disease Elimination, [1]). Even where effective vaccines or treatments exist, disease elimination presents an ongoing challenge. For example, after the establishment of the Global Malaria Eradication Program in 1955 by the World Health Organisation (WHO) it was later abandoned in 1969 due to funding shortages and drug resistance [2], leading to re-emergence of disease in Europe [3]. Assessing when a disease is close enough to elimination to die out without further intervention, thus prompting the end of a control campaign, is a problem of global economic importance. If campaigns are stopped prematurely it can result in disease resurgence and subsequently put control efforts back by decades. Conversely, the threat posed by newly emerging diseases such as SARS, Ebola or the recent COVID-19 pandemic strains available resources, places restrictions on global movement and disrupts the world’s most vulnerable societies. Identifying which newly-emerging diseases will present a global threat, and which will never cause a widespread epidemic is of critical importance.

To overcome the challenges identifying disease elimination or emergence, numerous studies have suggested the use of early warning signals (EWS) [4–9]. EWS are statistics that may be derived from data that change in a predictable way on the approach to a critical threshold. In epidemiology this threshold is commonly described as the point at which the basic reproduction number, *R*_0_, passes through *R*_0_ = 1. A system with *R*_0_ increasing through 1 describes an emerging disease whereas *R*_0_ decreasing through 1 results in disease elimination. We seek to find EWS to identify when a disease is approaching such a transition. We may identify such statistics using critical slowing down (CSD) theory, which indicates the imminent approach of a threshold, arising from slower recovery times from perturbations as a system approaches a critical transition [10, 11]. The recovery time increases when the dominant eigenvalue of the steady state passes through zero, since the eigenvalue determines the relaxation time of the system. As a result, increased recovery times can lead to stronger fluctuations around the steady state, causing variance around the steady state to increase. Additionally, a rising memory manifests itself as the rate of change of the system decreases to zero as a critical transition is approached, this results in the state of the system becoming more like its past state, i.e. autocorrelation increases. Therefore general signatures of critical slowing down include an increase in recovery time, variance and autocorrelation as a system nears a critical transition.

EWS offer the ability to anticipate a critical transition indirectly in real world noisy time series data, by observing, for example, increasing variance in the fluctuations around the steady-state [11, 12]. Statistical indicators offer a computationally inexpensive and efficient method for assessing the status of an infectious disease, presenting a simple mechanism for disease surveillance and monitoring of control policies.

The development of EWS is an active area of research in many fields, identifying the statistical signatures of abrupt shifts in many dynamical systems. Studies have applied EWS to historical data or laboratory experiments where a tipping point is known [10, 13, 14]; developed methods for using spatial variation [15, 16], explored the effects of detrending [7, 17] using the composition of multiple EWS [14, 18, 19]; and developed understanding of the limitations of EWS [20–22].

Discrepancies in statistical signatures have been discovered in a variety of historical datasets known to be going through a critical transition: from climate systems to stock markets, to applications with ecological field data [13, 20, 23]. These studies observed unexpected characteristic traits of common EWS, such as identifying a decreasing trend in variance or standard deviation, leading to a discussion on the robustness of indicators. It is therefore highly important to understand analytically how EWS are expected to change on the approach to a critical transition for different data types to avoid any misleading results.

The initial development of EWS in epidemiology focused on prevalence data, producing analytical solutions and numerically testing the capabilities for statistical indicators of emergence and elimination of infectious diseases [4–7]. Analysis of computer simulations of well-studied epidemiological systems have highlighted challenges such as seasonality [6] or detrending of epidemiological time series data [7]. However epidemiological data is typically collected in the form of the number of new infectious cases (incidence data) over a certain period of time (weekly/monthly/yearly). Generally, the exact date of infection or recovery of an individual is not known and therefore the exact number of infectious individuals at each point in time (the prevalence data that has been analysed) is unknown.

Simulation-based studies exploring incidence-type data have suggested that the potential for emergence of an infectious disease can be informed by statistical signatures [8, 9]. These studies represent the first attempts to understand the robustness of some indicators when used with disease emergence incidence data, subject to underreporting and time aggregation. Both studies find that EWS do precede disease emergence even when reporting is low. When the numerical performance of 10 EWS are compared, Brett *et al*. find that the mean and variance perform well unless incidence is subject to a highly overdispersed reporting error and they compare these results with previously studied prevalence results. Theoretical predictions are given for prevalence data, however the analytical behaviour of incidence is not explored.

O’Dea *et al*. [9] incorporate an observation model into a Birth-Death-Immigration (BDI) process to present an analytical study of EWS of disease emergence. This model allows prediction of the behaviour of EWS for dynamics captured by a BDI process but is not suitable for diseases with population-level immunity. O’Dea *et al*. additionally conducted an investigation into reporting errors in incidence-type data by recording the removal of individuals (“death” component in the BDI process). They describe the probability of a case being reported with either a Binomial or Negative Binomial distribution, allowing for over and under-reporting. In contrast to Brett *et al*., they conclude that the mean, variance and coefficient of variation (CV) are poor indicators since they are sensitive to reporting errors and insensitive to differences between transmission and recovery rates.

In this paper, we advance the current literature to describe generalised signatures of statistical indicators for incidence data, on the approach to a threshold, highlighting the differences between EWS descriptors of incidence and prevalence. Our results demonstrate that EWS of emergence exhibit an increasing variance, a trait associated with CSD and supporting results from Brett *et al*. and O’Dea *et al*. Strikingly however, we demonstrate that as a disease approaches elimination the opposite is true—variance decreases, and thus an increase in the variance of incidence is not observed as an early warning signal of eradication under the CSD framework.

Nevertheless we find that the time series trends of incidence are still a valuable tool to predict disease elimination. The discrepancy between prevalence and incidence on the one hand, and elimination and emergence on the other, could lead to potential problems in detecting thresholds if the differences are not clearly understood.

We introduce an analytical theory from stochastic processes to address why variance in incidence decreases for disease elimination. We study multiple other indicators of disease elimination predicted by this theory, and compare their responses with stochastic simulations. We also consider the rate of incidence as a measurement that can be extracted from incidence data. Notably, we find that on the approach to a critical transition the rate of incidence exhibits typical CSD signatures which correspond with prevalence data, such as an increasing variance. We present a broad analytical framework for EWS of incidence and rate of incidence for a variety of different disease systems. We explore more intricate systems where elimination is driven by different factors to understand the robustness of this theory. This simple generalised result can be applied to many infectious diseases undergoing emergence or elimination, a promising development for EWS of infectious diseases.

## Methods & mathematical theory

In this paper we focus on the application of EWS to disease elimination where there is a limited understanding on how time series statistics of incidence data behaves on the approach towards this threshold. We consider two simple models, where disease elimination is forced with different mechanisms, to explore how EWS of disease elimination behave for prevalence and incidence data. To demonstrate the broadness of our results, we additionally present a comparative case study to the analytical results for emerging diseases by O’Dea *et al*.

In this section we review the following models: SIS model (Susceptible-Infected-Susceptible model, see for example Keeling & Rohani [24]); SIS model with vaccination and SIS model with external force of infection. For each model that we have chosen to investigate, we derive the stochastic differential equations (SDEs) that describe the analytical behaviour of prevalence in these systems. Derivations of the analytical results and calculations of each statistic can be found in the supporting text (S1 Text). We present our analysis for incidence data and derive the corresponding statistical indicators. We exploit the well known fact that a counting process can be described by a Poisson process. We apply this result to the field of EWS to incorporate statistical signatures of a Poisson distributed variable to describe the behaviour of the number of new infectious cases in epidemiology.

We verify our analytical results for prevalence and incidence with simulated studies, and compare the contrasting results between prevalence and incidence. We measure the change in trend of multiple statistical indicators using the Kendall’s Tau score which gives an indication of an increasing or decreasing trend.

### Elimination and emergence models

#### SIS with social distancing

We begin with a simple example of a system that is approaching elimination from an existing endemic state of *I*. We consider an SIS model where the effective contact rate *β* acts as the control parameter. Effective reduction of *β* can be induced by public health campaigns (such as washing hands or improving food hygiene) and through social distancing (such as school closure). By decreasing *β*(*t*) in time, it slowly forces $R_{0}=\frac{\beta (t)}{\gamma }$ through the critical transition at *R*_0_ = 1. The transition probabilities for these dynamics are given in Table 1, where *β*(*t*) changes slowly in time, given by,

$$\begin{array}{ccc} \beta (t) & = & \beta_{0}\left(1-pt\right), \end{array}$$ (1)

$$\begin{array}{ccc} R_{0}\left(t\right) & = & \frac{\beta (t)}{\gamma }, \end{array}$$ (2)

and we fix the population such that *N* = *S* + *I*. Previously work has shown that the fluctuations, *ζ*, about the prevalence steady state, $\phi \left(t\right)=\frac{\langle I(t)\rangle }{N}$ can be separated using the linear noise approximation [4, 7, 25], see S1 Text for details and the corresponding SDE.

**Table 1:** Transition probabilities in prevalence theory for all models.

Event	Transition	Rate
**SIS with social distancing**		
Infection	*T*(*I* + 1|*I*)	$\frac{\beta (t)(N-I)I}{N}$
Recovery	*T*(*I* − 1|*I*)	*γI*
**SIS with vaccination**		
Infection	*T*(*S* − 1, *I* + 1|*S*, *I*)	$\frac{\beta_{0}SI}{N}$
Recovery	*T*(*S* + 1, *I* − 1|*S*, *I*)	*γI*
Incoming to *S* (non-vaccinated)	*T*(*S* + 1, *I*|*S*, *I*)	*μN*(1 − *p*(*t*))
Removal from *I*	*T*(*S*, *I* − 1|*S*, *I*)	*μI*
Removal from *S*	*T*(*S* − 1, *I*|*S*, *I*)	*μS*
**SIS emergence**		
Infection	*T*(*I* + 1|*I*)	$\frac{\beta (t)(N-I)I}{N}+\nu \left(N-I\right)$
Recovery	*T*(*I* − 1|*I*)	*γI*

#### SIS with increasing vaccination coverage

We consider an SIS model where a proportion of susceptible individuals are vaccinated and gain immunity to the disease, while the remaining (unvaccinated) individuals enter the susceptible compartment. By increasing the proportion of individuals vaccinated *p*(*t*), this control will reduce the effective reproduction number as the susceptible populations is depleting. Births and deaths are considered to allow for a non-zero steady state of *I* initially, and to ensure that the susceptible population does not decrease to zero. By increasing the proportion of individuals vaccinated *p*(*t*), the system is pushed away from this steady state. We gradually increase the proportion of vaccinated individuals over time by,

$$p(t)=p_{0}+pt,$$ (3)

$$R_{0}\left(t\right)=\frac{\left(1-p\left(t\right)\right)\beta_{0}}{\gamma +\mu },$$ (4)

to push the system through the critical transition at *R*_0_ = 1. We interpret the dynamics of the fluctuations about prevalence *I* and susceptible individuals *S*, with a two-dimensional Fokker-Planck Equation (see supplementary text S1 Text and Table 1 for transition rates).

#### SIS with external force of infection and increasing transmission

Finally we consider the SIS model with external infection which has been used to investigate EWS in prevalence and in incidence [4, 9]. We demonstrate how our analytical results compare for this system, and illustrate differences when applied to disease elimination.

In this model, in addition to the underlying SIS dynamics, susceptible individuals can be infected by an external force of infection (governed by parameter *ν*) that does not depend on the level of infection.

We consider the model in a stochastic formulation, with transition probabilities given in Table 1. Disease emergence is driven by increasing the effective contact rate *β*(*t*) over time, that slowly increases *R*_0_ through the critical transition at *R*_0_ = 1,

$$\beta (t)=\beta_{0}\left(1+pt\right),$$ (5)

$$R_{0}\left(t\right)\approx \frac{\beta (t)}{\gamma }.$$ (6)

Prior work by O’Regan & Drake [4] derived the SDE for the fluctuations, *ζ* about the prevalence steady state for this system—we have included them in the S1 Text for the convenience of the reader.

### Incidence theory

A counting process can be used as a generalised theory to understand the dynamics of the number of new events over a period of time. In particular, a diverse range of data types can be described by a counting process and this motivates us to characterise how statistics of such processes behave on the approach to a critical transition. Incidence (the number of new cases, *n*_*c*_) is a counting process, which is known to be described by a non-homogeneous Poisson process {*n*_*c*_(*t*): *t* ∈ [0, ∞)} with time dependent rate λ(*t*),

$$\begin{array}{ccc} n_{c}\left(t+\Delta t\right) & = & Poi(\int_{t}^{t+\Delta t}\lambda \left(s\right)ds)\approx Poi\left(\Delta t\lambda \left(t\right)\right). \end{array}$$ (7)

where the integral approximation holds for Δ*t* sufficiently small. In the supporting text (S1 Fig) we demonstrate that for our parameters, this approximation works well for Δ*t* up to 3. We can derive EWS in disease incidence aggregated over a time interval Δ*t* (e.g. daily, weekly, biweekly cases) using the well-known central moments of the Poisson distribution:

$$Var(n_{c})\approx \Delta t\lambda (t),$$ (8)

$$CV(n_{c})\approx \frac{1}{\sqrt{\Delta t\lambda (t)}},$$ (9)

$$SK(n_{c})\approx \frac{1}{\sqrt{\Delta t\lambda (t)}},$$ (10)

$$KT(n_{c})\approx \frac{1}{\Delta t\lambda (t)}.$$ (11)

Prior work from O’Dea *et al*. [9] & Brett *et al*. [8] have incorporated under-reporting using a negative binomial distribution; this can be included in this model when the rate λ(*t*) is itself a random variable. In particular, if λ(*t*) is distributed as a gamma distribution then Poi(λ) would be a negative binomial distribution. The gamma distribution is described by its mean and dispersion parameter (Θ). O’Dea *et al*. & Brett *et al*. took the mean to be *ξn*_*c*_, where *ξ* is the probability of reporting a case and considered different values for the dispersion parameter, relating to levels of overdispersion in the data.

Without under-reporting the rate of new cases is given by the incoming transition probabilities to the infectious state,

$$\begin{array}{c} \lambda (t)=T(I+1|I). \end{array}$$ (12)

A common form of this force of infection is,

$$\begin{array}{c} T\left(I+1|I\right)=\frac{\beta (t)S(t)I(t)}{N}, \end{array}$$ (13)

as such, λ(*t*) depends on the prevalence of infection, *I*(*t*). When we consider social distancing measures, *β*(*t*) is a function of time whereas for our vaccine uptake model *β*(*t*) = *β*_0_ is fixed. Infection can also be increased in other ways such as an external force of infection, $\lambda \left(t\right)=\frac{\beta (t)S(t)I(t)}{N}+\nu S\left(t\right)$, that is typically used to describe zoonotic spillover events or as an approximation for human migration.

We evaluate the statistical indicators of incidence, e.g. the variance (in incidence) given by Δ*t*λ(*t*) = Δ*tβ*(*t*)*S*(*t*)*I*(*t*)/*N*, by substituting in the solution to the ordinary differential equations of *I*(*t*) and *S*(*t*) (described by the mean field *σ*(*t*), see Table 2 and S1 Text).

**Table 2:** Model notation and parameter values shared among all models.

**Parameter**	**Description**	**Value**
*β*_0_	Initial Transmission Rate	$\beta_{0}^{\left\{1,2\right\}}=1,\beta_{0}^{\left\{3\right\}}=0.12$
*γ*	Recovery rate	*γ*^{1,3}^ = 0.2, *γ*^{2}^ = 0.18
*μ*	Population turn over rate	*μ*^{2}^ = 0.02
*p*_0_	Initial vaccination rate	$p_{0}^{\left\{2\right\}}=0$
*p*	Rate of change of *β*_0_ or *p*_0_	*p*^{1,2,3}^ = 0.002
*ν*	External rate of infection	*ν*^{1,2}^ = 0, *ν*^{3}^ = 0.001
*N*	Population Size	*N* = 10, 000
Δ*t*	Time aggregation of incidence data	Δ*t* = 1, daily
*T*	Time simulations run for	*T* = 500 (after burn in of 300 days)
BW	Bandwidth for RoI approx. simulations	*BW* = 30
**Model Notation**	**Description**	
*ζ*^{1},{3}^	Fluctuations about the infected steady state	
*ζ*_1_, *ζ*_2_	Fluctuations about the susceptible and infected steady state respectively (vaccination model)	
*ϕ*(*t*)	Proportion of infected individuals (mean-field)	
*ψ*(*t*)	Proportion of susceptible individuals (mean-field)	
*σ*(*t*) = *β*(*t*)*ϕ*(*t*)*ψ*(*t*)	Mean-field equation of the rate of incidence	
*n*_*c*_(*t*)	Number of new cases at time *t*	
λ(*t*)	Rate of Incidence (rate of the Poisson process)	
*η*	Fluctuations about the Rate of Incidence steady state	
Ext	Extinction Simulations (social-distancing & vaccination)	
Emg	Emergence Simulations	
Fix	Simulations with fixed parameters (null)	

values in braces directs to the model number which was implemented at that value. Superscript 1: SIS with social distancing; superscript 2: SIS with vaccination; superscript 3: SIS emergence. Parameters without braces are shared amongst all models.

We compare our approximation of incidence using a counting process with the recent study by O’Dea *et al*. [9]. In this work, the SIS model with external infection events was approximated with a Birth-Death-Immigration process, where an immigration event approximates the external force of infection; birth events give new infections and the death component is analogous with recovery events. O’Dea *et al*. derive statistics for incidence data by monitoring the number of individuals recovering (e.g. the transition rate *T*(*I* − 1|*I*) = *γI*). Results from this study can be found in the supplementary text (S1 Text). One limitation of this methodology is its difficulty to extend to other systems. It was developed for a specific disease emergence model—prompting the current search for generic EWS that can describe all epidemiological systems by using the easy-to-obtain transition probabilities.

### Rate of incidence theory

We also consider the rate of incidence (or the rate of the Poisson process) λ(*t*) = *T*(*I* + 1|*I*), which can be described dynamically with an SDE. Our analyses shows that the critical transition of the rate of the Poisson process corresponds to prevalence models (e.g. at *R*_0_ = 1) and importantly exhibits behaviours associated with CSD.

We investigate here calculating statistics on the rate of incidence (RoI) and its potential to be used as an EWS for disease transitions. Below we present our analytical results describing statistical indicators for each model. These theoretical solutions can be used to derive time-varying indicators for the fluctuations of the rate of incidence. Full derivations of the analytical work are given in the supporting text: S1 Text.

#### SIS: Social distancing and emergence

For the SIS model with social distancing (decreasing transmission) and the SIS model for emergence (increasing transmission), we describe the rate of incidence as $\lambda_{t}=\beta \left(t\right)\frac{(N-I)I}{N}+\nu \left(N-I\right)$, where for the former model *ν* = 0 as there are no external infections. We are interested in the statistical indicators of the rate of incidence, as such we substitute the linear noise approximation of *I*(*t*) (considered previously for prevalence data) and *S*(*t*). In particular, by considering the time derivative of λ_*t*_ we can conclude that the fixed points of the rate of incidence can be described by the transcritical bifurcation at *R*_0_ = 1. We find that the stability of the fixed points of λ_*t*_ also correspond to those of *I*, as expected.

We describe the fluctuations, *η*, about the steady state of $\lambda_{t}=\frac{\beta (N-I)I}{N}+\nu \left(N-I\right)$ using the linear noise approximation (LNA). We are interested in statistics calculated on the fluctuations about the rate of incidence, to develop new indicators of disease elimination (emergence). We derive the resulting analytical solution for *η* using Ito’s Change of variable formulae (details in supporting text: S1 Text) to approximate *η* with the following Gaussian process:

$$\lambda_{t}=N\sigma +N^{1/2}\eta ,$$ (14)

η≈[β(1-2ϕ)-νϕ)]ζ, (15)

$$\begin{array}{ccc} d\eta & = & [\beta \left(1-2\phi \right)-\gamma -\nu -\frac{2\beta }{\beta (1-2\phi )-\nu }\left(\beta \phi \left(1-\phi \right)-\gamma \phi +\nu \left(1-\phi \right)\right)]\eta dt\\ & + & \left(\beta \left(1-2\phi \right)-\nu \right)\sqrt{\beta \phi (1-\phi )+\gamma \phi +\nu (1-\phi )}dW_{t}. \end{array}$$ (16)

In particular, the changing behaviour of the variance of the rate of incidence as the system approaches disease elimination can be calculated from the SDE Eq 16,

$$\begin{array}{ccc} \frac{d\langle \eta^{2}\rangle }{dt} & = & 2(\beta \left(t\right)\left(1-2\phi \right)-\gamma -\nu -\frac{2\beta }{\beta (1-2\phi )-\nu }\left(\beta \phi \left(1-\phi \right)-\gamma \phi +\nu \left(1-\phi \right)\right))\left\langle \eta^{2}\right\rangle \end{array}$$ (17)

$$\begin{array}{ccc} & + & {\left(\beta \left(t\right)\left(1-2\phi \right)-\nu \right)}^{2}\beta \phi \left(1-\phi \right)+\gamma \phi +\nu \left(1-\phi \right). \end{array}$$ (18)

#### SIS with vaccination

If we consider models where there is population-level immunity, then $\lambda_{t}=\frac{\beta SI}{N}$ and we can no longer reduce the dimension of incoming transitions using *S* = *N* − *I*. This can be seen in the SIS model with increasing rate of vaccination, in particular the prevalence analysis of these systems presented in S1 Text results in a multivariable Fokker-Plank Equation.

However, we can similarly describe the fluctuations, *η*, about the steady state of $\lambda_{t}=\frac{\beta SI}{N}$ using the linear noise approximation (LNA) as with the above case.

We again use Ito’s change of variable formulae for the multivariable system (which depends on the fluctuations about susceptible and infectious individuals, *ζ*_1_ and *ζ*_2_ respectively) to approximate *η*. This leads to an SDE equation which depends on the description of *ζ*_1_ and *ζ*_2_ (Supplementary eqn. 25). In particular, we are interested in statistics of the rate of incidence, such as the variance, which can be simplified in terms of the original covariance matrix Θ (Supplementary eqn. 28) and mean-field equations of infectious (*ϕ*) and susceptible (*ψ*) individuals to give,

$$\lambda_{t}=N\sigma +N^{1/2}\eta ,$$ (19)

$$\eta \approx \beta (\phi \zeta_{1}+\psi \zeta_{2})),$$ (20)

$$\frac{Var(\eta )}{N}=\beta^{2}(\psi^{2}\Theta_{22}+\phi^{2}\Theta_{11}+2\phi \psi \Theta_{12}).$$ (21)

### Simulated study

#### Gillespie simulations

We use the Gillespie algorithm [26] to simulate each model, using time varying parameters (*β*(*t*) for SIS with social distancing & SIS emergence and *p*(*t*) for SIS with vaccination) to drive the model either to extinction (social distancing & vaccination) or emergence. We record prevalence outputs at time steps of 0.1 per day and we aggregate incidence outputs to daily time steps Δ*t* = 1. Parameters common to each model are given in Table 2. For SIS with social distancing, the transmission parameter *β* was reduced from *β*_0_ = 1 to 0, slowly forcing *R*_0_ = 5 to 0. For SIS with vaccination, the rate of vaccination was increased from *p*_0_ = 0 to 1, slowly forcing *R*_0_ = 5 to 0. For emergence, the transmission parameter *β* was increased from *β*_0_ = 0.12 to 0.24 so that the basic reproduction number increases from *R*_0_ ≈ 0.6 to ≈ 1.2.

Code to reproduce the simulations and calculate the statistical indicators is available online at https://github.com/ersouthall/Rate-Of-Incidence-EWS. A description of the numerical estimators used in this paper are listed in Supplementary S1 Table.

#### Numerical estimation of rate of incidence

A drawback of using the rate of incidence (RoI) as a measure of disease elimination, is the need to develop methods to extract this rate from incidence data. In our simulation study, we calculate the RoI in two ways from Gillespie output: *true RoI* and *rolling RoI*. After estimating RoI from the either method, we calculate the EWS of RoI over multiple realisations.

***True RoI***.

Firstly, using simulations of prevalence and taking the product $\frac{\beta (t)SI}{N}$ (or $\frac{\beta (t)SI}{N}+\nu S$ to include external infections), evaluates our definition of λ(*t*). This method, although unrealistic as it requires knowledge of prevalence (*I*), demonstrates the accuracy of the analytical results, as it is the “true” definition of RoI.

***Rolling RoI***.

An alternative method uses the Poisson property of incidence, illustrating that the rate of incidence λ(*t*) is equal to the mean and the variance of incidence over time. Our second method evaluates RoI by calculating the mean on a rolling window of the Gillespie output of incidence (*n*_*c*_) with bandwidth size *BW*. Likewise, we could also calculate the variance on a rolling window of the Gillespie output of incidence—we do not present this method here.

Taking the rolling average of incidence over time gives an approximation of the mean number of new cases (mean of the Poisson Process is RoI, λ(*t*)) for each realisation, we refer to this method as “rolling” RoI.

#### Calculation of statistical indicators

For each model, we also perform simulations where the disease has not fully gone through a critical transition (null model) which we refer to as Fix simulations. Fix simulations are a null model which has no control mechanism and the disease fluctuates about the fixed endemic steady state, at *R*_0_ = 5 (elimination models) and *R*_0_ = 0.5 (emergence models).

Before calculating the time changing statistics, we detrend each simulation by removing the mean over all realisations of that setting (Ext, Emg or Fix). We are interested in five common statistical indicators: variance (V), coefficient of variation (CV), skewness (SK), kurtosis (K) and autocorrelation lag-1 (AC(1)). We illustrate how EWS change over time, and how accurate the theory is to predicting these trends. Initially, we compare the analytical results of incidence, prevalence and RoI to the simulations by calculating each statistic over multiple realisations. In the section below, we describe how we calculate each statistic (over a moving window) to perform the receiver operating characteristic analysis for each detrended simulation of: incidence, prevalence and “rolling” RoI.

#### Kendall-tau score and receiver operator characteristic curves

The Kendall-tau score gives a measure of an increasing or decreasing trend of each statistic over the time series. We use the measure to evaluate whether a statistic corresponds to an increasing or decreasing trend and compare this for different data types (prevalence, incidence and RoI). The Kendall-tau score is defined as [27],

$$\begin{array}{c} \tau =\frac{\#\text{concordant}\;\text{pairs}-\#\text{discordant}\;\text{pairs}}{M(M-1)/2}, \end{array}$$ (22)

where *M* is the number of time points. Two points in the time series $\left(t_{1},x_{t_{1}}\right)$ and $\left(t_{2},x_{t_{2}}\right)$ with *t*_1_ < *t*_2_ are said to be a concordant pair if $x_{t_{1}}<x_{t_{2}}$, and a discordant pair if $x_{t_{1}}>x_{t_{2}}$. If the two points are equal ($x_{t_{1}}=x_{t_{2}}$) then the pair is neither concordant or discordant.

We compare the Kendall-tau scores calculated on simulations going through a critical transition with null simulations. We quantify the predictive power of each statistical indicator using its time-changing trend to classify simulations as either extinct (Ext simulations), emerging (Emg simulations) or null simulations (Fix simulations). We calculate each statistic on a moving window (size 50) for each detrended simulation, and compare the Kendall-tau score calculated over each time series up to two end points: before the critical transition (*t*_1_) and after the critical transition (*t*_2_).

We use receiver operating characteristic (ROC) analysis [28] to classify each simulations as either null or disease-changing and present a ROC curve (in Supplementary S10 Fig) which gives a graphical plot of the true/false positive rate for each statistical indicator. We compare each statistical indicator’s ability to correctly distinguish which Kendall-tau scores belong to those from a null simulation and which belong to a model undergoing a critical transition. The performance of each model statistic is given by the area under the curve (AUC) of the ROC curve.

The AUC score gives a predictive measure between different indicators, which we use to assess their performances. Good statistics have an AUC score close to 1 or 0 since this indicates the statistic is far from picking by chance. The closer the AUC score is to 0.5, the worse the statistical indicator is at identifying a critical transition. This is analogous to randomly selecting simulations that are the null and disease-changing simulations. A score close to 1 indicates nearly perfect sensitivity and specificity. For each EWS, we assume that an increasing trend represents a disease going through a critical transition. As a result a AUC score of 1 informs us that the indicator is increasing and that it is possible to identify all Ext/Emg simulations when compared to the null simulations by its increasing trend. An AUC score of 0 demonstrates that the time series trend is instead decreasing and as such it does not correspond to the predetermined prediction. A perfectly diagnosed decreasing indicator when compared to the null model will result in zero sensitivity under these conditions and an AUC score of 0.

## Results

### Variance (incidence and prevalence)

Variance is one of the most intuitive statistical indicators. As a system approaches a critical transition the time taken to recover from small perturbations increases, as described by Critical Slowing Down theory. This can be observed in the fluctuations about the steady state, which on the approach to a critical transition take longer to return and consequently vary far more, defining the increasing nature of variance as an early warning signal.

We evaluate analytical solutions of the variance in prevalence using the derived SDE for each model (SIS with social distancing: Supp. Eqn. 8, SIS with vaccination: Supp. Eqn.28, SIS emergence: Supp. Eqn. 30). We compare this to theoretical solutions of the variance in incidence given in Eq 8. The approximation that λ(*t*) = *β*(*t*)*ϕψ* + *νψ* ≈ *γϕ* was used by O’Dea *et al*., [9] and has been implemented in the wider literature; for this reason we also include this approximation for the rate of the Poisson Process describing incidence in the Supplementary Material S2 Fig.

**Fig 1:**
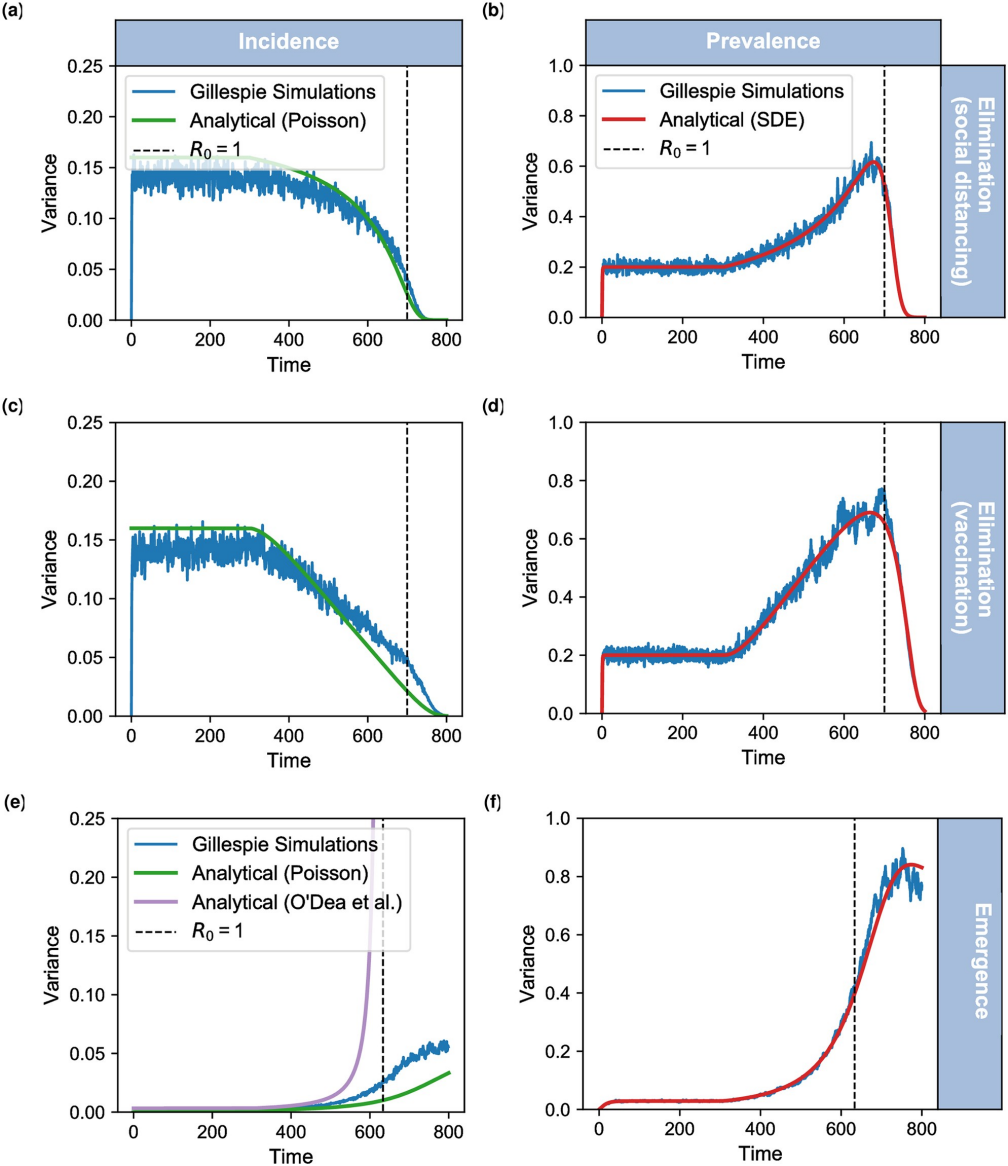
Comparing predictions to simulations for variance. For each model (SIS with social distancing; SIS with vaccination; SIS emergence) we calculate the variance between 500 homogeneous realisations at every time step (daily). Each figure shows: Poisson process distribution (green line); dynamic predictions (red line) and Gillespie simulations (blue line). The bottom left panel also shows the dynamical prediction from O’Dea *et al*. which was derived for this specific system (lilac line).

Fig 1 presents the simulated statistics for both prevalence and incidence theories of elimination and emergence, where we have plotted the variance between multiple homogeneous simulations at each time point (described in supporting text: S1 Table and with the null model: S5 Fig). Our prediction for the variance is similar to the stochastic simulations with a slight underdispersion in the incidence simulations (Fig 1a, 1c and 1e), since the theory (rate of Poisson process, green line) is higher than that of the variance in the fluctuations of the simulations (blue line). We note that our solution for SIS emergence (Fig 1e) follows the gradient of the stochastic simulations more closely. Since the analytical solution by O’Dea *et al*. (orange line) is evaluated at the steady state then the result diverges at the critical transition. It can be shown that for larger values of *β*_0_, O’Dea *et al*. results fit closer to the stochastic simulation, although the general trend of variance for both approaches follows the simulations.

We observe that variance in prevalence simulations (Fig 1b, 1d and 1f) increases on the approach to the critical transition, as predicted by critical slowing down. In comparison the variance in incidence decreases before the critical transition for all disease elimination models (SIS with social distancing Fig 1a and SIS with vaccination Fig 1c) and increases similarly to prevalence for the disease emergence model (Fig 1e).

As expected by our Poisson process analysis, the variance of this system should be the same as the mean of the system. Therefore for disease elimination models, we should expect a decreasing variance (along with a decreasing mean) when calculated on incidence data, in contrast to an increasing variance with prevalence data. Likewise with disease emergence models we expect an increasing variance to correspond to the increasing mean. This demonstrates that our analysis of incidence has successfully predicted the time-varying variance for these different systems.

### Variance (rate of incidence)

Fig 1 demonstrates that the variance of incidence does not necessarily increase on the approach to a critical transition. A new approach for working with incidence-type data is to consider the rate of incidence, λ(*t*) = *T*(*I* + 1|*I*), which for each model we have derived the dynamical SDE (see Methods).

**Fig 2:**
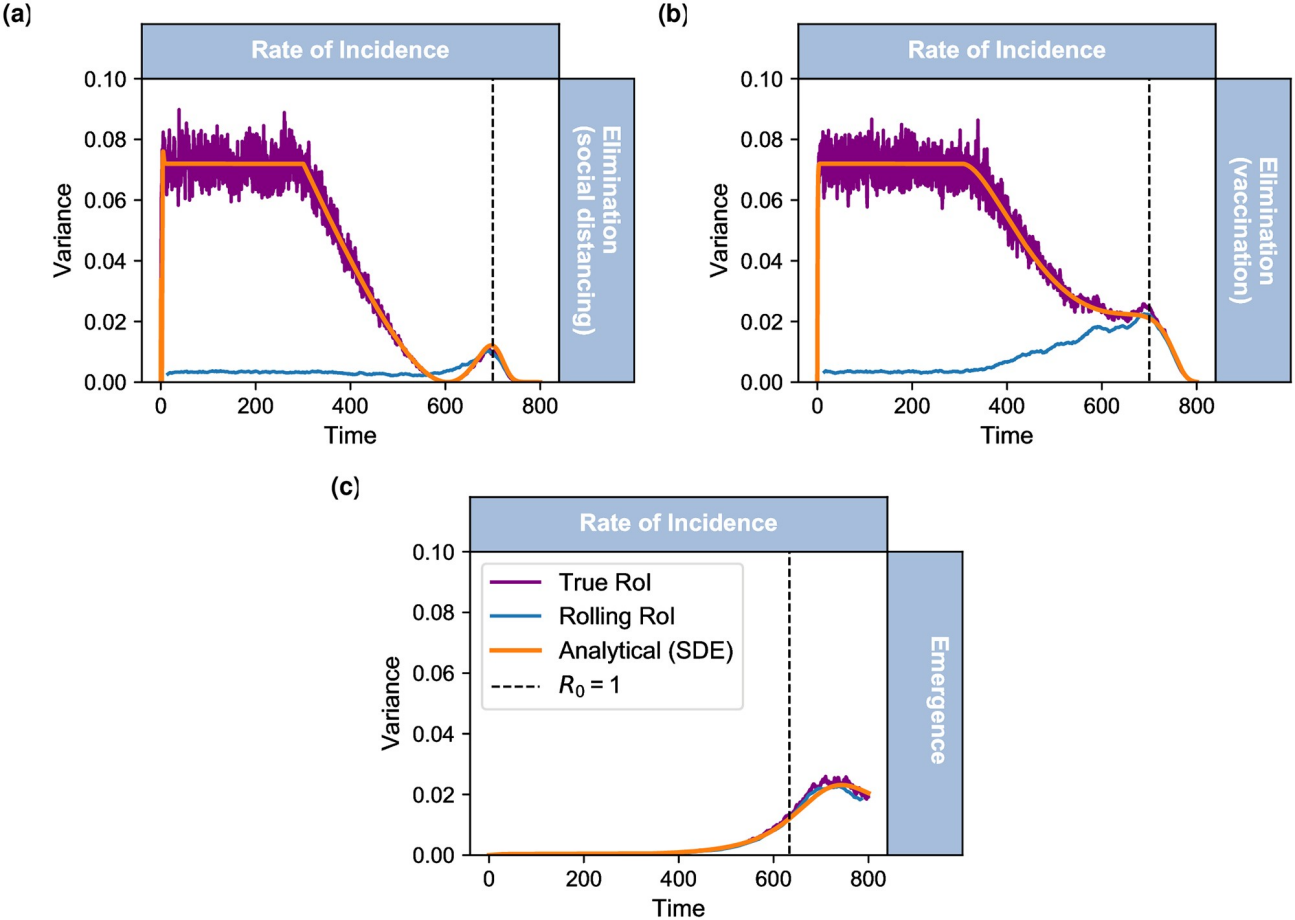
Variance calculated on the rate of incidence. For each model (SIS with social distancing (panel a); SIS with vaccination (panel b); SIS emergence(panel c)) we calculate the variance on the rate of incidence, RoI between 500 homogeneous realisations at every time step (daily). Each figure shows: dynamic solution (orange line); rolling RoI calculated from new cases *n*_*c*_ (blue line) and true RoI calculated from the production of prevalence, susceptible and effective contact rate ($\lambda \left(t\right)=\frac{\beta SI}{N}$, purple line).

We present results calculated in RoI simulations using the two methods: “true” and “rolling” RoI, in Fig 2. The first method uses prevalence data (“true”, purple line) and corresponds well with the analytical solution (orange line) for all models and the latter method (smoothing incidence data “rolling”, blue line) fits particularly well for the emergence model (Fig 2(c)). However it does not follow as closely to some time-varying properties of the variance for elimination scenarios (Fig 2(a) and 2(b)) respectively. Although the early dynamics are misrepresented for disease elimination, all time series indicate an increasing variance on the approach to the critical transition.

We observe that the analytical prediction fits well with the stochastic simulations of “true” RoI ($\frac{\beta SI}{N}$, purple line Fig 2(a) and 2(b)) for SIS with social distancing and SIS with vaccination respectively. This demonstrates that this theory approximates the behaviour of the system well. Indeed, we observe that approximating the rate of incidence by smoothing Gillespie simulations of new cases (“rolling” RoI, blue line Fig 2(a) and 2(b)) predicts a similar increasing behaviour before the critical transition. This corresponds to the same peak as the analytical prediction and “true” simulations. However, it fails to capture the magnitude of the behaviour earlier on in the dynamics.

An area that still needs to be addressed with the “rolling” RoI methodology is understanding why the early dynamics in the disease elimination scenarios are poor. In the supporting text S3 Fig, we demonstrate that if the disease is approaching elimination at a slower rate, both methods (“true” and “rolling”) converge to the analytical solution. We chose parameters such that *β*(*t*) changes on a much slower time scale and approaches disease elimination (social distancing model) at the same rate as *β*(*t*) approaches disease emergence for the SIS emergence scenario (*R*_0_ changes from 1.2 to 0, $\beta_{0}^{\left\{1\right\}}=0.24$). As the system changes slowly enough then the system will be approximately ergodic, such that the moving average resembles the mean incidence. Thus the “rolling” method will be closer to the “true” solution. In comparison, the faster a system changes over time, will correspond to a wider range in incidence cases across the moving window. Resulting in a lower mean over the window which can be seen in Fig 2(a) and 2(b); although the statistic will be more pronounced at the threshold.

We also investigated determining a suitable window size for calculating “rolling” RoI. In the supporting text S4 Fig, we considered a large range of bandwidth sizes: from window size 10 to 125 (for a total time period of size 800) and took *BW* = 30 in the main text. We found that the “peak” as elimination is approached was pronounced and captured for all bandwidth choices. We find it reassuring that the methodology is robust for bandwidth size choice, however all choices failed to reproduce the magnitude of the early dynamics. This limitation could result in misinterpretation when used in practice.

We find that for SIS with vaccination (Fig 2(b)) the general trend of the variance is less pronounced at the critical transition than observed for SIS with social distancing. We observe that the analytical solution (Fig 2(b) orange line) and true stochastic simulations (Fig 2(b) purple) only slightly increase before the critical transition, implying this trend would be difficult to detect in real-world data. In particular, the Kendall-tau score which can be an indication of an increasing trend, is negative (decreasing, *τ* = −1) for this model, whilst for SIS with social distancing and SIS emergence we find that *τ* = 0.987 and *τ* = 1 respectively. Although, we observe that the “rolling” simulations of the rate of incidence (Fig 2(b) blue line) exhibit similar properties as SIS with social distancing. We again observe that the early stage dynamics of this method have not predicted the expected behaviour of the analytical solution. It can be noted that *R*_0_ decreases at the same rate as the SIS with social distancing model, suggesting that this could also be due to when *R*_0_ is not slowly changing.

In Fig 2(c) we observe that both measurements of the variance of λ_*t*_ calculated on stochastic simulations of SIS emergence have closely followed the analytical solution of variance. As expected the true stochastic simulations (Fig 2(c) purple line) follow closely to the theory, supporting that this derivation of *η* is correct. More interestingly, calculating the variance of the rate of incidence directly from simulations of new cases (*n*_*c*_, Fig 2(c) blue line) has performed far better than when presented in elimination models (Fig 2(a) and 2(b)). For emergence, we observe that the variance of the rate of incidence increases before the critical threshold, similar to prevalence for this model. We further found that the early dynamics of the “rolling” RoI simulations represent the true behaviour of the variance. This result may be due to *R*_0_ increasing more slowly in emergence model than the rate it decreases at in the elimination models, satisfying the ergodic condition.

### Other statistical indicators

In this section, we investigate the potential of identifying an epidemiological transition using five commonly implemented early-warning signals: variance, coefficient of variation (CV), skewness, kurtosis and lag-1 autocorrelation (AC(1)). Exploration of each EWS follows similarly to variance, as analysed above theoretically and numerically for prevalence, incidence and rate of incidence. In the supporting text, time series trends for each indicator are presented for each dataset and model (S5 Fig: variance, S6 Fig: CV, S7 Fig: Skewness, S8 Fig: kurtosis, S9 Fig: AC(1)), along with analytical analyses for these indicators (S1 Text).

**Fig 3:**
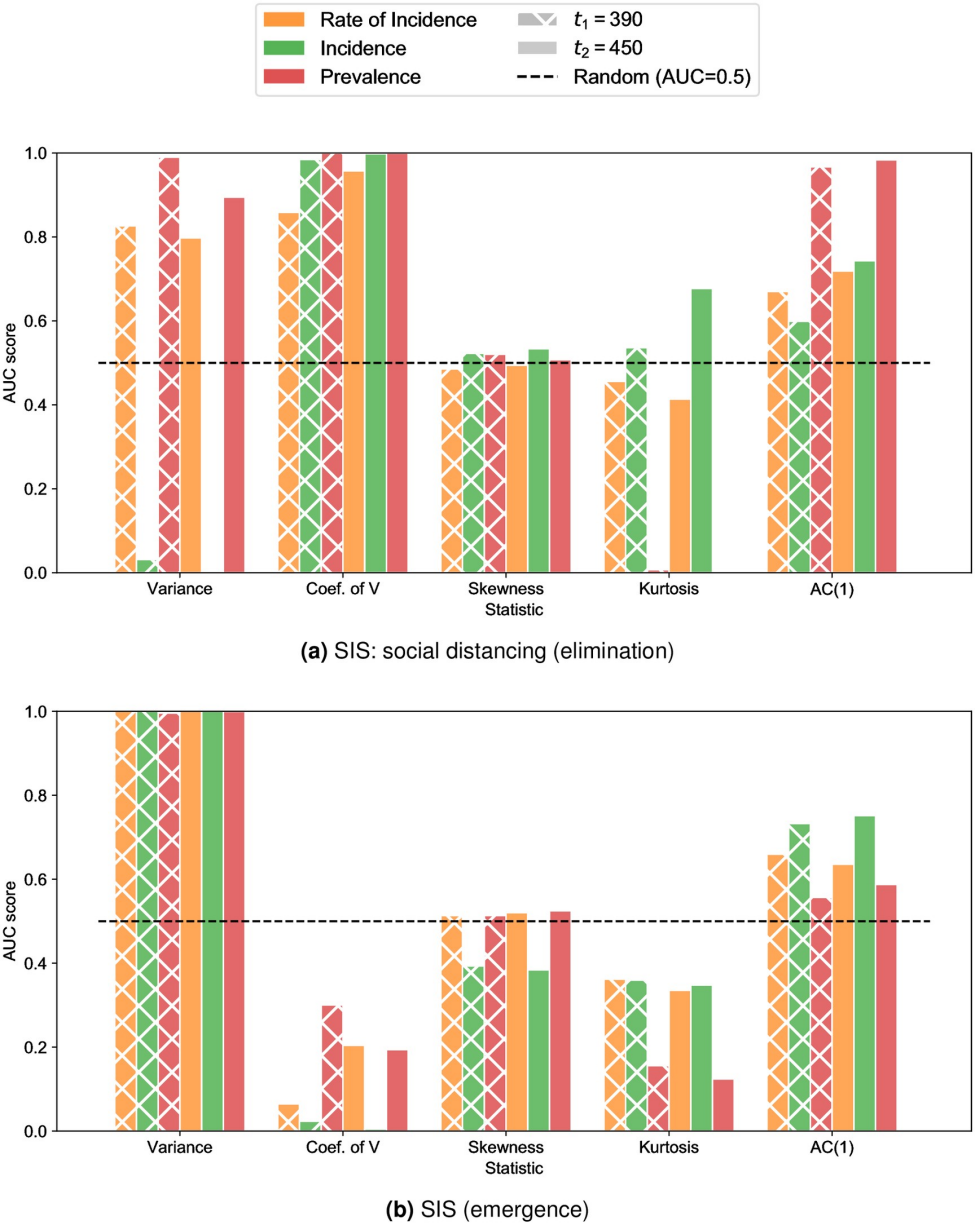
AUC scores for different EWS. We compare the performance of 5 common statistical indicators for SIS with social distancing (a) for disease elimination and SIS emergence (b). The Rate of Incidence is taken to be the “rolling” RoI.

Here, we quantify these time series trends for each statistical indicator using the Kendall-Tau score as a measure of an overall increasing or decreasing trend. We present in Fig 3 the predictive power of each statistical indicator by its measuring the AUC score up to two end points: before the critical transition (*t*_1_) and after the critical transition (*t*_2_) which gives an overall score of the true/false positive rate.

Fig 3 highlights which indicators are in some cases increasing (AUC close to one), decreasing (AUC close to zero) or are poor indicators (AUC close to 0.5). In particular, as discussed in the previous section, variance always increases prior to disease emergence (Fig 3(b)). However, for disease elimination (SIS with social distancing: Fig 3(a) and SIS with vaccination: S11 Fig) results are substantially different when we compare variance calculated in rate of incidence and prevalence (orange and red bars respectively) with incidence (green bars). For RoI and prevalence data types, the statistical signature is an increasing variance with an AUC near 1. This is in contrast to the latter where the trend is decreasing with an AUC near 0. However, the results for variance (both increasing and decreasing) are highly predictive (|*AUC* − 0.5| ≈ 0.5). Thus, if a system is not known or there is difficultly in determining the type of data, incorrect conclusions could be drawn when interpreting the time series trend.

We observe that skewness is a poor indicator because of its inability (AUC score close to 0.5) to identify disease elimination with any type of disease data it is applied to (rate of incidence, incidence and prevalence). Identifying emergence with skewness in prevalence or RoI data (red and orange bars respectively) is also very poor and its predictive ability is only slightly increased with incidence (green bars). Whereas, coefficient of variation calculated on all types of disease data (rate of incidence, incidence and prevalence) and for both SIS elimination models, exhibits a near perfect ability to identify the increasing trend.

## Discussion

While studies for EWS on incidence-type data have been growing in recent years, theoretical exploration of how these indicators change on the approach to a critical transition have been neglected. In this paper, we have shown that the typical trends of EWS that precede a critical transition are exhibited in prevalence-type data but do not always exist in incidence-type data. In particular, we have focused our investigation on the trend of variance over time as an infectious disease system approaches a tipping point.

Prior work has shown that variance in incidence increases on the approach towards disease emergence. However, our work highlights that this property might not be a result from critical slowing down theory as first expected. We have shown it is a consequence of the counting process that can approximate incidence-type data. As such, we demonstrated that the variance in incidence is expected to follow the mean in incidence. In particular, the variance of incidence will increase on the approach to disease emergence, but will notably decrease before a disease elimination threshold. We applied these findings to two systems of disease elimination and verified that variance of incidence exhibits a decreasing trend on the approach, following the behaviour of the mean of incidence, instead of rising.

Therefore, it is highly recommended to understand analytically how EWS change on the approach to a critical transition in order to avoid misleading results. The generalised theory of a counting process can be applied to many other systems outside of the scope of epidemiology where we would expect a decreasing variance preceding a critical transition. Potential applications include the observation of animals through camera traps, disease surveillance sampling in wildlife or movements in stock prices, which are all examples of incidence-type data. Notably, a substantial number of studies on ecosystem data, climate data and financial data have observed inconsistencies in statistical indicators [13, 20, 23, 29]. In particular, systems where a rising variance but decreasing autocorrelation is exhibited [11, 20] or recent work finding both decreasing variance and decreasing autocorrelation for systems where the basin of attraction narrows as the critical transition is approached [30], are examples which do not produced CSD based warning signals. Although we found the Poisson process to be overdispersed in the context of epidemiology, it provides a broad framework which can be extended to many other infectious disease systems using the incoming transition probabilities into the infectious class.

We proposed extracting the rate of incidence (RoI) or intensity of Poisson process from incidence-type data to illustrate that utilising CSD, such as observing an increasing variance, could depend on suitable data which directly undergoes a bifurcation. In particular, we have shown that the critical threshold in the RoI corresponds with that of prevalence; and as expected we demonstrated that the trend in variance in RoI does increase before an imminent epidemiological transition. A clear limitation with using RoI is developing suitable methods for extracting this quantity from incidence data. We presented a method (named “rolling” RoI) to perform this extraction and found it poorly represented the early dynamics of RoI. However, the signal correctly increased prior to the critical transition in correspondence with the theory and this trend was consistently exhibited for a large range of bandwidth choices. Future work will include developing these methods to approximate RoI from real-world data.

We applied five early warning signals to simulated datasets comprising of the three discussed data types: prevalence, incidence and rate of incidence. The simulated data we have investigated represents perfect reporting or the “best case scenario”. Often is the case that there is underreporting that may reduce the detectability of signals in real-world data. The work we have presented here can be extended to include a gamma distributed intensity λ. Using a gamma distributed rate of incidence will account for reporting errors as described by O’Dea *et al*.

Overall, our study suggests that a robust indicator is one that shares a highly predictive time series trait (|*AUC* − 0.5| ≈ 0.5) amongst all three data types, even with inconsistent trends (increasing or decreasing). Therefore, we suggest that variance and coefficient of variation are overall good indicators due to their high predictive power in all cases. Coefficient of variation is a robust indicator for disease elimination since the trend is similar between different types of data (Fig 3(a)) and S11 Fig. However discrepancies are demonstrated when considering opposite disease thresholds as shown with disease emergence (Fig 3(b)) which has a decreasing trend for CV and performs less well with disease prevalence data.

However, we found that kurtosis and AC(1) are not robust indicators. Although kurtosis and AC(1) have a predictive trend with prevalence data, this is not typically the data which is readily available. In particular, kurtosis is highly predictive (with a decreasing trend) in prevalence data on the approach to disease elimination (Fig 3(a)) and fairly predictive with an decreasing trend in the case of prevalence with emergence (Fig 3(b)); it is a poor indicator for all other types of data. Likewise, although AC(1) has a clear increasing trend for prevalence data elimination systems (Fig 3(a), S11 Fig), it is less predictive trend for incidence and RoI data. Additionally, the trend is not distinct for any datasets when considering an emergence transition, therefore there is a potential for this indicator to be used incorrectly. In the cases where an EWS is poor in some types of data but good for others could lead to misleading judgements of systems, and therefore are not robust.

These findings support prior work on prevalence and initial work from O’Dea *et al*. and Brett *et al*. with incidence-type data. Our analytical exploration of incidence has indicated a new data source, RoI, which can be extracted from incidence timeseries. A potential powerful tool would be to compute variance and CV indicators with different types of data (incidence, rate of incidence and prevalence) and ensemble these. A composite of multiple statistical indicators was suggested by Drake & Griffen [14] and has been applied to case studies with the same data-type and a combination of EWS by Kefi *et al*. [19] to help interpret between different critical transitions and also has successfully detected transitions using an ensemble of different time series data [18]. This suggests a potential approach to achieve a single metric from a combination of indicators calculated on multiple timeseries data with different trends, such as we have observed with incidence and RoI, to achieve a more pronounced indication of disease transitions.

Additionally, further work would be to include a heterogeneous ensemble as suggested by O’Dea *et al*. [9], whereby all parameters are sampled randomly for each realisation rather than being equal. This will lead to more realistic results, as each parameter sample represents time series data from different locations, as suggested by studies on spatial statistics, a promising method for addressing limited data [7, 15, 16]. Comparatively, we have shown here that computing the statistics on a homogeneous ensemble although unrealistic, it returns exact stochastic behaviours of the system and we used this to verify the simulated study with the theory.

In conclusion, there is a tremendous potential for using early warning signals to provide evidence on our progress towards elimination and inform public health policies. We have indicated that by monitoring simple statistics over time it is possible to observe disease emergence and elimination, which with further development offers a promising solution for an automated system that can update time series statistics in real-time as new data becomes available. This would be particularly useful for emerging diseases where EWS could be used to prompt early detection and help aid rapid responses. The focus of our paper has provided insight on how statistics behave for different types of infectious disease data, where we considered suitable data which could be incorporated into such monitoring system. We have researched the resemblance of observed time series results between different data types, a necessary exploration for the development of EWS before they can impact decision making. We reported that some indicators traits are inconsistent across all data types and some EWS differ significantly between disease thresholds: elimination and emergence. Knowledge of the type of data which has been collected is imperative to avoid misleading judgements in response to time series trends. Our work has provided analytical evidence to understand why results differ, improving our ability to monitor EWS for infectious disease transitions.

## Supporting information

S1 Text Analytical derivations. (PDF)

S1 Table List of early warning signals. (PDF)

S1 Fig Sensitivity to time aggregation (Δ*t*) when approximating non-homogeneous Poisson process. For each model (Model 1 (panel a and b): SIS with social distancing (elimination); Model 2 (panel c and d): SIS increasing vaccination (elimination); Model 3 (panel e and f): SIS increasing transmission (emergence) we calculate the variance between 500 homogeneous realisations subject to aggregation of time steps (Δ*t*). We take the average of new cases over time step aggregation. Each figure shows: theory (dotted black line); critical transition (dashed black line) and Gillespie simulations (colours correspond to time aggregation). The last model also shows the dynamical prediction from O’Dea which was derived for this specific system (solid black line).
(PDF)

S2 Fig Approximating incidence by using the rate of the Poisson Process, λ(*t*) = Δ*tγI*. For each model: SIS with social distancing (a), SIS with vaccination (b), SIS increasing transmission/emergence (c) and the SIR epidemic curve (d) we plot two approximations of the intensity of a Poisson process. The dashed line is the point where *R*_0_ = 1 or *R*_*eff*_ = 1 (effective reproduction number—SIR model). Blue line is variance calculated over stochastic simulations. Orange line: Poisson Process with λ(*t*) = *γI*(*t*). Green line: Poisson Process with λ(*t*) = *βS*(*t*)*I*(*t*). Panel (d): SIR epidemic curve, is included here to demonstrate an example where the approximation *βSI*/*N* ≈ *γI* isn’t appropriate. This is the SIR model with: *β*(*t*) = 0.1 and *γ* = 0.01.
(PDF)

S3 Fig Slower rate reduces discrepancies between theory and simulated variance in “rolling” RoI. SIS with social distancing (elimination) and parameters *β*_0_ = 0.24. In this example, the dynamics follow those of Section 1.1 but with different parameters. This leads to the approach towards *R*_0_ = 1 being at a slower rate where *R*_0_ changes from 1.2 to 0.
(PDF)

S4 Fig Approximation method “rolling” RoI is consistent across different bandwidth sizes. Calculating “rolling” RoI with a range of bandwidths for: a). SIS with social distancing, b). SIS with vaccination and c). SIS emergence.
(PDF)

S5 Fig Comparing predictions to simulations for variance. For each model: a,b,c).SIS social distancing (elimination); d,e,f). SIS increasing vaccination (elimination); g, h, i). SIS increasing transmission, (emergence) we calculate the variance between 500 homogeneous realisations at every time step (daily). Each figure shows: Poisson Process distribution (green line); dynamic predictions (red line) and Gillespie simulations (Ext and Emg, blue line). The last model also shows the dynamical prediction from O’Dea which was derived for this specific system (orange line).
(PDF)

S6 Fig Comparing predictions to simulations for coefficient of variation. For each model: a, b, c). SIS social distancing (elimination); d,e,f). SIS increasing vaccination (elimination); g,h,i). SIS increasing transmission, (emergence) we calculate the CV between 500 homogeneous realisations at every time step (daily). Each figure shows: Poisson Process distribution (green line); dynamic predictions (red line) and Gillespie simulations (Ext and Emg, blue line).
(PDF)

S7 Fig Comparing predictions to simulations for skewness. For each model: a,b,c). SIS social distancing (elimination); d,e,f). SIS increasing vaccination (elimination); g,h,i). SIS increasing transmission, (emergence) we calculate the skewness between 500 homogeneous realisations at every time step (daily). Each figure shows: Poisson Process distribution (green line); dynamic predictions (red line) and Gillespie simulations (Ext and Emg, blue line).
(PDF)

S8 Fig Comparing predictions to simulations for Kurtosis. For each model: a,b,c). SIS social distancing (elimination); d,e,f). SIS increasing vaccination (elimination); g,h,i). SIS increasing transmission, (emergence) we calculate the kurtosis between 500 homogeneous realisations at every time step (daily). Each figure shows: Poisson Process distribution (green line); dynamic predictions (red line) and Gillespie simulations (Ext and Emg, blue line).
(PDF)

S9 Fig **S9A: Comparing predictions to simulations for autocorrelation lag-1** For each model: a,b,c). SIS social distancing (elimination); d,e,f). SIS increasing vaccination (elimination); g,h,i). SIS increasing transmission, (emergence) at each point *t* we calculate the lag-1 AC on an interval [*t* − *W*/2, *t*+ *W*/2]. We then calculate the mean of this AC over 500 homogeneous realisations at every time step. Each figure shows: Poisson Process distribution (green line); dynamic predictions (red line) and Gillespie simulations (Ext and Emg, blue line). **S9B: Evaluating the MSE between theoretical prediction and simulations for different window sizes**. For each mode, we calculate how the error between theoretical solution and simulations changes with different window sizes used in the moving average calculated of AC(1). We demonstrate that these errors narrow, and define the minimum window size of which the error is below some threshold (given in the plots) to be chosen suitable window size (for the moving average calculations).
(PDF)

S10 Fig ROC curves for indicators: V, CV and AC(1) (Figo. S10A); Skewness and Kurtosis (Figo. S10B). Comparison of disease transitioning simulations: SIS with social distancing (solid lines); SIS with vaccination (dashed lines) and SIS disease emergence with increasing transmission (dotted lines) to the null model (Fixed endemic simulations). Each curve compares the null simulations to each model for different indicators and for each data type: prevalence (P, red lines), incidence (I, green lines) and rate of incidence (RoI, orange lines). ROC curves calculated for indicators: variance; coefficient of variation (CV) and lag-1 autocorrelation (AC). Kendall-tau scores measured over each data-type up to *t*_1_ = 390 (pre disease transmission) are given in the left column and up to *t*_2_ = 450 (post disease transmission) are given in the right column. For each ROC curve we measured the AUC score which is given in legend, which is an indication of how predictive each indicator is by its ability to distinguish between elimination simulations and the null model.
(PDF)

S11 Fig AUC scores for different EWS (SIS with vaccination). We compare the performance of 5 common statistical indicators for the SIS model: disease elimination with vaccination. For each ROC curve we measured the AUC which is an indication of how predictive each indicator is by its ability to distinguish between elimination simulations and the null model. The closer to 0.5 signifies the worst performance (random diagnosis). We evaluate the Kendall-tau score up to before the critical transition (*t*_1_ = 390) and after the critical transition (*t*_2_ = 450) which gives an indication if the EWS is increasing or decreasing. A score of 1 demonstrates that it is possible to identify all Ext simulations when compared to null simulations by its increasing trend (i.e. perfect sensitivity, true positive rate). A score of 0 means that there is zero sensitivity and instead the simulations are decreasing.
(PDF)
