# Supplementary material for: Prospects for detecting early warning signals in discrete event sequence data: Application to epidemiological incidence data
Source: PLoS Comput Biol. 2020 Sep 22;16(9):e1007836. doi: 10.1371/journal.pcbi.1007836 (PMC7531856; doi:10.1371/journal.pcbi.1007836)
Supplement: S1 Text — (PDF) [file pcbi.1007836.s001.pdf]

Contents

1 Analytical Derivations **1**
1.1 SIS with social distancing . . . 1
1.1.1 Prevalence . . . 1
1.1.1.1 Variance . . . 3
1.1.1.2 Coefficient of Variation . . . 3
1.1.1.3 Skewness . . . 4
1.1.1.4 Kurtosis . . . 4
1.1.1.5 lag-τ autocorrelation . . . 4
1.1.2 Rate of Incidence . . . 6
1.1.2.1 Variance . . . 7
1.1.2.2 lag-τ autocorrelation . . . 7
1.2 SIS with vaccination . . . 8
1.2.1 Prevalence . . . 8
1.2.1.1 lag-τ autocorrelation . . . 9
1.2.2 Rate of Incidence . . . 10
1.2.2.1 Variance . . . 10
1.2.2.2 lag-τ autocorrelation . . . 11
1.3 SIS Emergence . . . 12
1.3.1 Prevalence . . . 12
1.3.1.1 Variance . . . 12
1.3.1.2 lag-τ autocorrelation . . . 12
1.3.2 Rate of Incidence . . . 13
1.3.2.1 Variance . . . 13
1.3.2.2 lag-τ autocorrelation . . . 14
1.3.3 Incidence results from O'Dea *et al.* . . . 14

1 Analytical Derivations

1.1 SIS with social distancing

1.1.1 Prevalence

The following section was derived in O'Regan & Drake (3) (setting $\eta = 0$ in Eqn. 1) and Dessavre (2), we present it again here for the ease of the reader. Schematic of the dynamics:

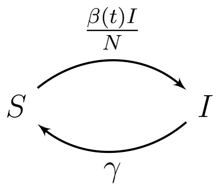

For the SIS model without external infections, the mean-field equations are given by $\frac{d\phi}{dt} = \beta\phi(1-\phi) - \gamma\phi$ where $\phi = \frac{\langle I \rangle}{N}$.

The following deviation follows from van Kampen (Chapter 8 and 10, (1)). The linear noise approximation (LNA) for the discrete infectious state I is given by:

$$I = N\phi(t) + N^{1/2}\zeta. \tag{1}$$

The general form of the master equation for the SIS model based on the transition probabilities given in Table 1 is,

$$\begin{aligned}\frac{dP(I,t)}{dt} &= T(I|I-1)P(I-1,t)+T(I|I+1)P(I+1,t)-T(I-1|I)P(I,t)-T(I+1|I)P(I,t)\\ &= \frac{\beta(t)\,(N-(I-1))\,(I-1)}{N}P(I-1,t)+\gamma(I+1)P(I+1,t)-\gamma IP(I,t)-\frac{\beta(t)(N-I)I}{N}P(I,t)\end{aligned} \tag{2}$$

The master equation can be written using step operators which act on an arbitrary function of n, defined as $\mathbb{E}f(n)=f(n+1)$ and $\mathbb{E}^{-1}f(n)=f(n-1)$.

$$\begin{aligned}\frac{dP(I,t)}{dt} &= \mathbb{E}^{-1}T(I+1|I)P(I,t)+\mathbb{E}T(I-1|I)P(I,t)-T(I-1|I)P(I,t)-T(I+1|I)P(I,t)\\ &= (\mathbb{E}^{-1}-1)T(I+1|I)P(I,t)+(\mathbb{E}-1)T(I-1|I)P(I,t)\\ &= (\mathbb{E}^{-1}-1)\frac{\beta(t)(N-I)I}{N}P(I,t)+(\mathbb{E}-1)\gamma IP(I,t) \qquad (3)\\ P(I,0) &= \delta_{I,I_0}.\end{aligned}$$

The step operators have a simple expansion involving powers of $N^{-1/2}\partial/\partial\zeta$. Since the operators take I to $I+1$ then it follows that it takes, $\zeta=\frac{I-N\phi(t)}{N^{1/2}}$ to$\frac{I+1-N\phi(t)}{N^{1/2}}=\zeta+\frac{1}{N^{1/2}}$. From here, we can perform a Taylor expansion and derive the following expression for $\mathbb{E}$:

$$\begin{aligned}\mathbb{E}f(\zeta) &= f(\zeta+N^{-1/2})\\ &= f(\zeta)+N^{-1/2}f'(\zeta)+\frac{1}{2}(N^{-1/2})^2f''(\zeta)+...\\ \mathbb{E} &= 1+N^{-1/2}\frac{\partial}{\partial\zeta}+\frac{1}{2}N^{-1}\frac{\partial^2}{\partial\zeta^2}+...\\ \mathbb{E}^{-1} &= 1-N^{-1/2}\frac{\partial}{\partial\zeta}+\frac{1}{2}N^{-1}\frac{\partial^2}{\partial\zeta^2}-...\end{aligned}$$

Define a new probability distribution function Π by $P(I,t)=\Pi(\zeta,t)$. The derivative of the probability distribution function with respect to t,

$$\frac{\partial P(I,t)}{\partial t}=\frac{\partial\Pi}{\partial\zeta}\frac{d\zeta}{dt}+\frac{\partial\Pi}{\partial t}=-N^{1/2}\frac{d\phi}{dt}\frac{\partial\Pi}{\partial\zeta}+\frac{\partial\Pi}{\partial t} \tag{4}$$

is needed for deriving the continuous space master equation.

Combining equations 3 and 4 together, we can write down the continuous space master equation:

$$\begin{aligned}-N^{1/2}\frac{d\phi}{dt}\frac{\partial\Pi}{\partial\zeta}+\frac{\partial\Pi}{\partial t} &= (\mathbb{E}^{-1}-1)T(I+1|I)P(I,t)+(\mathbb{E}-1)T(I-1|I)P(I,t)\\ &= (-N^{-1/2}\frac{\partial}{\partial\zeta}+\frac{1}{2}N^{-1}\frac{\partial^2}{\partial\zeta^2}-...)T(I+1|I)\Pi(\zeta,t)\\ &+ (N^{-1/2}\frac{\partial}{\partial\zeta}+\frac{1}{2}N^{-1}\frac{\partial^2}{\partial\zeta^2}+...)T(I-1|I)\Pi(\zeta,t)\\ &\qquad \text{and substitute the linear approximation}\\ &\approx (-N^{1/2}\frac{\partial}{\partial\zeta}+\frac{1}{2}\frac{\partial^2}{\partial\zeta^2})\beta(1-\phi-N^{-1/2}\zeta)(\phi+N^{-1/2}\zeta)\Pi(\zeta,t)\\ &+ (N^{1/2}\frac{\partial}{\partial\zeta}+\frac{1}{2}\frac{\partial^2}{\partial\zeta^2})\gamma(\phi+N^{-1/2}\zeta)\Pi(\zeta,t). \qquad (5)\end{aligned}$$

We collect powers of N in equation 5 and substitute the mean-field deterministic approximation as $N \to \infty$ (macroscopic description which ignores fluctuations). This results in the linear Fokker-Planck equation for this system:

$$\begin{aligned}\frac{\partial \Pi}{\partial t} &= N^{1/2}(\beta\phi(1-\phi)-\gamma\phi)\frac{\partial \Pi}{\partial \zeta} + (N^{1/2}\frac{\partial}{\partial \zeta} + \frac{1}{2}\frac{\partial^2}{\partial \zeta^2} + ...)\gamma(\phi + N^{-1/2}\zeta)\Pi(\zeta,t) \\ &+ (-N^{1/2}\frac{\partial}{\partial \zeta} + \frac{1}{2}\frac{\partial^2}{\partial \zeta^2} - ...)(\beta\phi(1-\phi) + \beta(1-2\phi)N^{-1/2}\zeta - \beta N^{-1}\zeta^2)\Pi(\zeta,t)\end{aligned}$$

we collect terms of order N^0,

$$\frac{\partial \Pi}{\partial t} = -(\beta(1-2\phi)-\gamma)\frac{\partial \zeta \Pi}{\partial \zeta} + \frac{1}{2}(\beta(1-\phi)\phi + \gamma\phi)\frac{\partial^2 \Pi}{\partial \zeta^2}.$$

Or equally we can represent this in terms of the corresponding SDE for ζ,

$$I = N\phi + N^{1/2}\zeta, \tag{6}$$

$$d\zeta = (\beta(1-2\phi)-\gamma)\zeta dt + \sqrt{\beta\phi(1-\phi)+\gamma\phi}dW_t. \tag{7}$$

1.1.1.1 Variance

The solution for the analytical variance can be deduced from the following equations,

$$\begin{aligned}\frac{\partial \langle \zeta^2 \rangle_t}{\partial t} &= \int_{-\infty}^{\infty} \zeta^2 \frac{\partial \Pi}{\partial t} d\zeta \\ &= -(\beta(1-2\phi)-\gamma)\int_{-\infty}^{\infty} \zeta^2 \frac{\partial \zeta \Pi}{\partial \zeta} d\zeta + \frac{1}{2}(\beta(1-\phi)\phi + \gamma\phi)\int_{-\infty}^{\infty} \zeta^2 \frac{\partial^2 \Pi}{\partial \zeta^2} d\zeta\end{aligned}$$

Then, integration by parts twice

$$\begin{aligned} &= 2(\beta(1-2\phi)-\gamma)\int_{-\infty}^{\infty} \zeta^2 \Pi d\zeta + (\beta(1-\phi)\phi + \gamma\phi)\int_{-\infty}^{\infty} \Pi d\zeta \\ \frac{\partial \langle \zeta^2 \rangle_t}{\partial t} &= 2(\beta - \gamma - 2\beta\phi)\langle \zeta^2 \rangle_t + \beta(1-\phi)\phi + \gamma\phi \\ &= N\frac{d\sigma^2}{dt} = (\beta - \gamma - 2\beta\phi)N\sigma^2 + \beta(1-\phi)\phi + \gamma\phi. \end{aligned} \tag{8}$$

At steady state when $\frac{d\phi}{dt} = 0$ and $\frac{d\sigma^2}{dt} = 0$, we obtain $\phi* = 1 - \frac{\gamma}{\beta}$ and $\sigma^2 * = \frac{1}{N}\frac{1}{R_0}$.

1.1.1.2 Coefficient of Variation

The coefficient of variation (CoV) represents the ratio of the standard deviation to the mean. The mean of the fluctuations can be found by taking moments (similar to variance) to achieve,

$$\frac{d\mu}{dt} = \frac{\partial \langle \zeta \rangle_t}{\partial t} = (\beta(1-2\phi)-\gamma)\langle \zeta \rangle_t, \tag{9}$$

$$\tag{10}$$

which solving equals to $\langle \zeta \rangle_t = \langle \zeta \rangle_0 exp(-\int \beta(1-2\phi) - \gamma dt)$. Since we initially have no fluctuations, we find that for all t, $\langle \zeta \rangle_t 0$ or in words the mean of the Gaussian Process is zero.

For this reason, we find the coefficient of variation of the un-detrended data of I to avoid the division by the zero mean in the fluctuations. From Ito's Formulae we can write the SDE of I and the following change in moments of $\langle I \rangle$ and $\langle I^2 \rangle$ to be,

$$dI = \left((\beta(1-2\phi)-\gamma)I + N\phi^2\beta\right)dt \tag{11}$$

$$+ \sqrt{\beta\phi(1-\phi)+\gamma\phi}dW_t \tag{12}$$

$$\frac{\langle I \rangle}{dt} = (\beta(1-2\phi)-\gamma)\langle I \rangle + N\phi^2\beta \tag{13}$$

$$\frac{\langle I^2 \rangle}{dt} = 2(\beta(1-2\phi)-\gamma)\langle I^2 \rangle + 2N\phi^2\beta\langle I \rangle + \beta(1-\phi)\phi + \gamma\phi \tag{14}$$

Coefficient of variation is defined as,

$$\frac{\sqrt{\langle I^2\rangle - \langle I\rangle^2}}{\langle I\rangle}.$$

1.1.1.3 Skewness

Since we have approximated ζ with a Guassian SDE, it follows that the Skewness ζ is zero. By considering the 3rd moments and the fact that $\langle\zeta\rangle_0 = 0$ and $\langle\zeta^3\rangle_0 = 0$ then,

$$\frac{d\mu}{dt} = \frac{\partial\langle\zeta\rangle_t}{\partial t} = (\beta(1-2\phi)-\gamma)\langle\zeta\rangle_t, \tag{15}$$

$$\langle\zeta\rangle_t = 0 \tag{16}$$

$$\frac{\partial\langle\zeta^3\rangle_t}{\partial t} = 3(\beta(1-2\phi)-\gamma)\langle\zeta^3\rangle_t + 3(\beta(1-\phi)\phi+\gamma\phi)\langle\zeta\rangle_t = 0 \tag{17}$$

However the Skewness of I is non-zero, we can solve the third moment of I to be,

$$\frac{d\langle I^3\rangle}{dt} = 3(\beta(1-2\phi)-\gamma)\langle I^3\rangle + 3N\phi^2\beta\langle I^2\rangle + 3(\beta(1-\phi)\phi+\gamma\phi)\langle I\rangle \tag{18}$$

where standardised skewness is defined as,

$$\frac{\langle I^3\rangle - 3\langle I^2\rangle\langle I\rangle + 2\langle I\rangle^3}{(\langle I^2\rangle - \langle I\rangle^2)^{3/2}}$$

1.1.1.4 Kurtosis

We consider the excess standardised kurtosis statistic, KT=$\frac{\langle\zeta^4\rangle}{\langle\zeta^2\rangle} - 3$ which standardises the kurtosis w.r.t the variance. Excess kurtosis (subtracting 3) is usually reported in order to make a Gaussian distribution have a kurtosis score of zero. The 4th moment of ζ can be found to be,

$$\frac{\partial\langle\zeta^4\rangle_t}{\partial t} = 4(\beta(1-2\phi)-\gamma)\langle\zeta^4\rangle_t + 6(\beta(1-\phi)\phi+\gamma\phi)\langle\zeta^2\rangle_t \tag{19}$$

1.1.1.5 lag-τ autocorrelation

We approach solving the analytical solution of ACF for each model about the quasi-stationary states using the power spectrum with the Wiener-Khinchin Theorem. The same result can also achieved (in a more convoluted integral) from the Fokker-Planck equation by multiplying through by $\zeta\zeta_0\Pi_0(\zeta)$ and computing the double integral.

$$d\zeta = (\beta(1-2\phi)-\gamma)\zeta dt + \sqrt{\beta\phi(1-\phi)+\gamma\phi}\,dW_t$$

$$\frac{d\zeta}{dt} = \zeta\lambda + Q^{1/2}\gamma(t)$$

where,

$$\lambda = \beta(1-2\phi)-\gamma$$

$$Q = \beta\phi(1-\phi)+\gamma\phi$$

$$\gamma(t) = \frac{dW_t}{dt}$$

Rearranging we get,

$$\zeta = \frac{1}{\lambda}\left[\frac{d\zeta}{dt} - Q^{1/2}\gamma(t)\right].$$

It suffices to perform a Fourier transformation of the Langevin equation:

$$\tilde{\zeta} = \int_{-\infty}^{\infty} \zeta(t) e^{-i\omega t} dt$$

Using the inverse Fourier Transformation $\zeta(t) = \frac{1}{2\pi}\int_{-\infty}^{\infty} \tilde{\zeta}(\omega) e^{i\omega t} d\omega$, we can write the Fourier Transform of ζ as

$$\begin{aligned}
\tilde{\zeta}(\omega) &= \int_{-\infty}^{\infty} \frac{1}{\lambda}\left[\frac{d}{dt}\left(\frac{1}{2\pi}\int_{-\infty}^{\infty} \tilde{\zeta}(\omega') e^{i\omega' t} d\omega'\right) - Q^{1/2}\gamma(t)\right] e^{-i\omega t} dt \\
&= \frac{1}{2\pi\lambda}\int_{-\infty}^{\infty} dt \left(\int_{-\infty}^{\infty} \left[i\omega'\tilde{\zeta} - Q^{1/2}\tilde{\gamma}\right] e^{it(\omega' - \omega} d\omega'\right) \\
&= \frac{1}{2\pi\lambda}\int_{-\infty}^{\infty} \left[i\omega'\tilde{\zeta} - Q^{1/2}\tilde{\gamma}\right] \delta(\omega' - \omega) d\omega'
\end{aligned}$$

The last line resulting from expressing the delta function as $\delta(x-a) = \frac{1}{2\pi}\int_{-\infty}^{\infty} e^{ip(x-a)} dp$ and $\int_{-\infty}^{\infty} f(t)\delta(t-T)dt = f(T)$.

Bringing this together we have,

$$\begin{aligned}
\tilde{\zeta} &= \frac{1}{\lambda}(i\tilde{\zeta}\omega - Q^{1/2}\tilde{\gamma}) \\
\tilde{\zeta} &= \frac{Q^{1/2}\tilde{\gamma}}{i\omega - \lambda}
\end{aligned}$$

The spectral density defines the factor of proportionality of the variance,

$$S(\omega) = \lim_{T\to\infty} \frac{1}{T}\langle \tilde{\zeta}(\omega)\tilde{\zeta}^*(\omega)\rangle$$

The Fourier transform of white noise $\tilde{\gamma}$ is only defined on finite intervals,

$$\begin{aligned}
\tilde{\gamma}(\omega) &= \Delta t \sum_{j=1}^{T/\Delta t} \gamma(t_j) e^{i\omega t_j} \\
t_j &= j\Delta t \\
\langle \tilde{\gamma}(\omega)^2 \rangle &= T
\end{aligned}$$

As such,

$$\begin{aligned}
S(\omega) &= \lim_{T\to\infty} \frac{1}{T}\langle \frac{Q\tilde{\gamma}^2}{\omega^2 + \lambda^2}\rangle \\
&= \frac{Q}{\omega^2 + \lambda^2}
\end{aligned}$$

The Wiener-Khinchin theorem states that the inverse transform of $S(\omega)$ is the autocovariance function $C(\tau)$ which offers a fast numerical estimate of the autocorrelation function: $C(\tau)/C(0)$,

$$\begin{aligned}
C(\tau) &= \lim_{T\to\infty} \frac{1}{T}\int_{-T/2}^{T/2} \zeta(t)\zeta(t+\tau) dt \\
&= \frac{1}{2\pi}\int_{-\infty}^{\infty} S(\omega) e^{i\omega\tau} d\omega
\end{aligned}$$

Notably, variance is given by $\langle \zeta^2 \rangle = \frac{1}{2\pi}\int_{-\infty}^{\infty} S(\omega) dw$. For this example,

$$\sigma^2 = \frac{1}{2\pi}\int_{-\infty}^{\infty} \frac{Q}{\omega^2+\lambda^2} d\omega$$
$$= \frac{Q}{2|\lambda|}$$

Lag-τ autocorrelation,

$$C(\tau) = \frac{1}{2\pi}\int_{-\infty}^{\infty} \frac{Q}{\omega^2+\lambda^2} e^{i\omega\tau} d\omega$$
$$= \frac{Q}{2|\lambda|} e^{-|\lambda|\tau}$$

In particular, lag-1 autocorrelation for prevalence $ACF = e^{-|\lambda|} = e^{-|\beta(1-2\phi)-\gamma|}$
So at steady state, lag-1 ACF is $e^{-|\beta-\gamma|}$.

1.1.2 Rate of Incidence

We define the rate of new infectious cases to be given by the incoming transition probability in class I,

$$\lambda(t) = T(I|I-1) = \frac{\beta SI}{N} = \frac{\beta(N-I)I}{N}$$

.

We are interested in the fluctuations about this rate, as such we similarly consider the LNA:

$$n_c = N\sigma + N^{1/2}\eta$$
$$\sigma = \beta\phi(1-\phi)$$

λ_t can also be written in terms of the fluctuations about I (defined by variable ζ above),

$$n_c = \beta SI/N$$
$$= N\beta\phi(1-\phi) + N^(1/2)\beta(1-2\phi)\zeta - \beta\zeta^2$$
$$\eta = \beta(1-2\phi)\zeta - \beta N^{-1/2}\zeta^2$$
Take the limit $N \to \infty$
$$\eta = \beta(1-2\phi(t))\zeta = f(\zeta, t)$$

We can use the previously derived results for the SDE describing $d\zeta$ with Ito's change of variable formula for a function dependent on t: $f(\zeta, t)$, to evaluate:

$$df(\zeta,t) = f_\zeta d\zeta + f_t dt + 1/2 f_{\zeta\zeta} b^2 dt$$
$$df(\zeta,t) = [\beta(1-2\phi)(\beta(1-2\phi)-\gamma)\zeta - 2\beta\zeta(\beta\phi(1-\phi)-\gamma\phi)]\, dt + \beta(1-2\phi)\sqrt{\beta\phi(1-\phi)+\gamma\phi} dW_t$$
$$= \left[\eta(\beta(1-2\phi)-\gamma) - 2\frac{\eta}{1-2\phi}(\beta\phi(1-\phi)-\gamma\phi)\right] dt + \beta(1-2\phi)\sqrt{\beta\phi(1-\phi)+\gamma\phi} dW_t$$

The Fokker-Planck equation which is equivalent to the above SDE is given by,

$$\frac{d\Pi(\eta,t)}{dt} = -\frac{\partial}{\partial\eta}\left((\beta(1-2\phi)-\gamma) - 2\frac{1}{1-2\phi}(\beta\phi(1-\phi)-\gamma\phi)\right)\eta\Pi(\eta,t)$$
$$+ \frac{1}{2}\frac{\partial^2}{\partial\eta^2}\left(\beta^2(1-2\phi)^2(\beta\phi(1-\phi)+\gamma\phi)\Pi(\eta,t)\right)$$

1.1.2.1 Variance

To give an analytical expression for the variance we can take moments using the FPE. In general, for a FPE of the form $\frac{d\Pi(x,t)}{dt} = -\frac{\partial}{\partial x}(a(y)x\Pi(x,t)) + \frac{1}{2}\frac{\partial^2}{\partial x^2}(b(y)\Pi(x,t))$ then the variance is given by $\frac{d\langle x^2\rangle}{dt} = 2a\langle x^2\rangle + b$, this can be shown using integration by parts (see above "Prevalence - Variance"). The variance of η is given by,

$$\frac{d < \eta^2 >}{dt} = 2\left((\beta(1-2\phi)-\gamma) - 2\frac{1}{1-2\phi}(\beta\phi(1-\phi)-\gamma\phi)\right) < \eta^2 > +\beta^2(1-2\phi)^2(\beta\phi(1-\phi)+\gamma\phi)$$

At steady state,

$$\langle\eta^2\rangle = \frac{\gamma}{\beta}(2\gamma-\beta)^2$$

1.1.2.2 lag-τ autocorrelation

It can be shown similarly as with prevalence, that we can use a Fourier Transform on the SDE equation of $d\eta$.

As such, we find that

$$\tilde{\eta} = \frac{Q^{1/2}\tilde{\gamma}}{i\omega - \lambda} \tag{20}$$

$$\lambda = \beta(1-2\phi) - \gamma - 2\frac{1}{1-2\phi}(\beta\phi(1-\phi)-\gamma\phi) \tag{21}$$

$$Q = \beta^2(1-2\phi)^2(\beta\phi(1-\phi)+\gamma\phi) \tag{22}$$

Then the lag-1 ACF is $e^{-|\lambda|} = e^{-|\beta(1-2\phi)-\gamma-\frac{2}{1-2\phi}(\beta\phi(1-\phi)-\gamma\phi|}$. Evaluated at steady state ($\phi^* = 1-\frac{\gamma}{\beta}$) both ACF lead to $e^{-|\beta-\gamma|}$.

1.2 SIS with vaccination

1.2.1 Prevalence

This work is being presented for the first time here. The schematic for this model is:

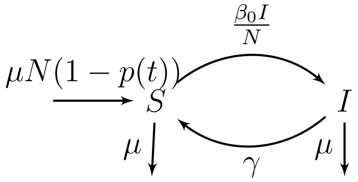

Following the same steps as the SIS model with social distancing, we derive for the SIS with vaccination model. As it is no longer true that $N = S + I$, since we will have an immune class following vaccination, this results in a Multivariate Fokker-Planck equation. The mean-field dynamics are described by a system of ODEs, where $\psi(t) = \frac{<S>}{N}$ and $\phi(t) = \frac{<I>}{N}$ and,

$$\frac{d\psi}{dt} = \mu(1 - p(t) - \psi) - \beta_0\psi\phi + \gamma\phi,$$
$$\frac{d\phi}{dt} = \beta_0\psi\phi - \phi(\mu + \gamma).$$

The critical point of the mean-field equations gives rise to the basic reproduction ratio, $R_0 = \frac{\beta_0(1-p(t))}{\mu+\gamma}$. We can describe the stochastic dynamics using the master equation with two step operators $\mathbb{E}_S, \mathbb{E}_I$ and the corresponding transition probabilities for these dynamics.

$$\begin{aligned}\frac{dP(S,I,t)}{dt} &= (\mathbb{E}_S\mathbb{E}_I^{-1} - 1)P(S,I,t)T(S-1,I+1|S,I)\\ &+ (\mathbb{E}_S^{-1} - 1)P(S,I,t)T(S+1,I|,S,I)\\ &+ (\mathbb{E}_S^{-1}\mathbb{E}_I - 1)P(S,I,t)T(S+1,I-1|S,I)\\ &+ (\mathbb{E}_I - 1)P(S,I,t)T(S,I-1|S,I).\end{aligned} \tag{23}$$

Equation 23 is non-linear and results in the N-expansion being necessary to determine variance in fluctuations.

The linear noise approximation anzats are taken to be

$$S = N\psi(t) + N^{1/2}\zeta_1,$$
$$I = N\phi(t) + N^{1/2}\zeta_2.$$

where ζ_1 defines the fluctuations about the susceptibles ($\psi = \frac{\langle S\rangle}{N}$) and ζ_2 defines the fluctuations about the infecteds ($\phi = \frac{\langle I\rangle}{N}$). We can define a new probability distribution $P(S,I,t) = \Pi((\zeta_1,\zeta_2),t)$ and substitute this into the master equation 23 to get:

$$\begin{aligned}&- N^{1/2}\frac{d\psi}{dt}\frac{\partial\Pi}{\partial\zeta_1} - N^{1/2}\frac{d\phi}{dt}\frac{\partial\Pi}{\partial\zeta_2} + \frac{\partial\Pi}{\partial t}\\ &= (\mathbb{E}_S\mathbb{E}_I^{-1} - 1)T(S-1,I+1|S,I)\Pi\\ &+ (\mathbb{E}_S^{-1}\mathbb{E}_I - 1)T(S+1,I-1|,S,I)\Pi\\ &+ (\mathbb{E}_S^{-1} - 1)T(S+1,I|S,I)\Pi\\ &+ (\mathbb{E}_S - 1)T(S-1,I|S,I)\Pi\\ &= (\mathbb{E}_S\mathbb{E}_I^{-1} - 1)\beta N(\psi + N^{-1/2}\zeta_1)(\phi + N^{-1/2}\zeta_2)\Pi(\zeta,t)\\ &+ (\mathbb{E}_S^{-1}\mathbb{E}_I - 1)(\gamma+\mu)N(\phi + N^{-1/2}\zeta_2)\Pi(\zeta,t)\\ &+ (\mathbb{E}_S^{-1} - 1)\mu N(1 - \psi - \phi - N^{-1/2}\zeta_1 - N^{-1/2}\zeta_2)\Pi(\zeta,t)\\ &+ (\mathbb{E}_S - 1)\mu Np(t)\Pi(\zeta,t).\end{aligned} \tag{24}$$

The multivariate Fokker-Planck Equation is fully described in terms of matrices A and B, where B is symmetric and positive definite. If both A and B are constant matrices then the solution is Gaussian (linear Fokker-Planck Equation),

$$\frac{\partial \Pi(\zeta, t)}{\partial t} = -\sum_{i,j}^{2} A_{ij} \frac{\partial}{\partial \zeta_i}(\zeta_j \Pi) + \frac{1}{2} \sum_{i,j} B_{ij} \frac{\partial^2 \Pi}{\partial \zeta_i \partial \zeta_j}. \tag{25}$$

A_{ij} and B_{ij} can be found by substituting the mean-field equations and collecting leading order and next to leading order terms. For the SIS model with vaccination the matrices are given by:

$$A = \begin{bmatrix} -\beta\phi - \mu & \gamma - \beta\psi \\ \beta\phi & \beta\psi - \mu - \gamma \end{bmatrix},$$
$$B = \begin{bmatrix} \beta\psi\phi + \mu(1 - \psi + p(t)) + \gamma\phi & -\phi(\beta\psi + \mu + \gamma) \\ -\phi(\beta\psi + \mu + \gamma)) & \phi(\beta\psi + \mu + \gamma) \end{bmatrix}.$$

Then the Gaussian solution is determined by the first and second moments, which are used to derive analytical solutions to important statistical indicators such as variance and coefficient of variation:

$$\partial_t \langle \zeta_k \rangle = \sum_j A_{kj} \langle \zeta_j \rangle, \tag{26}$$
$$\partial_t \langle \zeta_k \zeta_l \rangle = \sum_i A_{ki} \langle \zeta_i \zeta_l \rangle + \sum_j A_{lj} \langle \zeta_k \zeta_j \rangle + B_{kl}. \tag{27}$$

We can combine equations 26 and 27 by defining the covariance matrix Θ, where each element equals, $\Theta_{ij} \langle\langle x_i x_j \rangle\rangle = \langle x_i x_j \rangle - \langle x_i \rangle \langle x_j \rangle$ and

$$\partial_t \Theta = A\Theta + \Theta A^T + B, \tag{28}$$

where variance of the infectious fluctuations, ζ_2, is given by Θ_{22}

1.2.1.1 lag-τ autocorrelation

The fluctuations of the system can be described by the equivalent form of the SDE:

$$\begin{bmatrix} \frac{d\zeta_1}{dt} \\ \frac{d\zeta_2}{dt} \end{bmatrix} = A \begin{bmatrix} \zeta_1 \\ \zeta_2 \end{bmatrix} + \begin{bmatrix} \Gamma_1(t) \\ \Gamma_2(t) \end{bmatrix}, \tag{29}$$

where $\Gamma_1(t)$ and $\Gamma_2(t)$ are white noise processes with the covariance matrix B. To analyse system 29 about the quasi-stationary approximation, we take the Fourier transformation of $A\underline{\zeta} = \frac{d}{dt}\underline{\zeta} - \underline{\gamma}$ where $\underline{\zeta} = (\zeta_1, \zeta_2)$ and $\underline{\gamma} = (\Gamma_1, \Gamma_2)$.

Then Fourier Transform (using the FT of a derivative, if $Y(t) = \frac{d^n}{dt^n}(X(t))$ then $Y(\tilde{\omega}) = (i\omega)^n X(\tilde{\omega})$),

$$i\omega \begin{bmatrix} \tilde{\zeta_1}(\omega) \\ \tilde{\zeta_2}(\omega) \end{bmatrix} = A \begin{bmatrix} \tilde{\zeta_1} \\ \tilde{\zeta_2} \end{bmatrix} + \begin{bmatrix} \Gamma_1 \\ \Gamma_2 \end{bmatrix}$$
$$\begin{bmatrix} \tilde{\zeta_1}(\omega) \\ \tilde{\zeta_2}(\omega) \end{bmatrix} = (i\omega I - A)^{-1} \begin{bmatrix} \Gamma_1 \\ \Gamma_2 \end{bmatrix}$$

Interested in the FT of the infectious noise term, ζ_1, to derive the Power Spectrum and from this the quasi stationary variance and autocorrelation. Then,

$$\tilde{\zeta_2}(\omega) = \frac{1}{d - \omega^2 + i\omega T} \left[a_{21}\Gamma_1(\omega) - (a_{11} - i\omega)\Gamma_2(\omega) \right]$$
$$d = det(A)$$
$$T = trace(A)$$

Power Spectrum is defined as,

$$
\begin{aligned}
S_{\zeta_2}(\omega) &= \langle \tilde{\zeta_2} \tilde{\zeta_2}^* \rangle \\
&= \frac{a_{21}^2 B_{11} - 2a_{21}a_{11}B_{12} + a_{11}^2 B_{22} + B_{22}\omega^2}{(d-\omega^2)^2 + T^2\omega^2}
\end{aligned}
$$

The autocovariance function $C(\tau)$ is defined below and in particular the variance is given by $C(0)$ and autocorrelation lag τ is given by $C(\tau)/C(0)$,

$$
\begin{aligned}
C(\tau) &= \frac{1}{2\pi} \int_{-\infty}^{\infty} S_{\zeta_2}(\omega) cos(\omega\tau) d\omega \\
\sigma^2 = C(0) &= \frac{1}{2\pi} \int_{-\infty}^{\infty} S_{\zeta_2}(\omega) d\omega
\end{aligned}
$$

1.2.2 Rate of Incidence

Similarly to SIS with social distancing, the rate of new infectious cases is defined by the incoming transition probability in class I,

$$
n_c = T(I|I-1) = \beta SI/N
$$

.

We are interested in the fluctuations about this rate, as such we similarly consider the LNA:

$$
\begin{aligned}
n_c &= N\sigma + N^{1/2}\eta \\
\sigma &= \beta\phi\psi
\end{aligned}
$$

For this model, n_c is written in terms of the fluctuations about I (defined by variable ζ_2 above) *and* the fluctuations of S (ζ_1),

$$
\begin{aligned}
n_c &= \beta SI/N \\
&= N\beta\phi\psi + N^(1/2)\beta(\phi\zeta_1 + \psi\zeta_2) + \beta\zeta_1\zeta_2 \\
\eta &= \beta(\phi\zeta_1 + \psi\zeta_2) + \beta N^{-1/2}\zeta_1\zeta_2 \\
& \text{Take the limit } N \to \infty \\
\eta &= \beta(\phi\zeta_1 + \psi\zeta_2)
\end{aligned}
$$

1.2.2.1 Variance

We are interested in the variance of the fluctuations of new cases: $\langle \eta^2 \rangle$. We can derive this in terms of elements of the Covariance matrix Θ,

$$
\begin{aligned}
\langle \eta^2 \rangle &= \langle \beta^2(\phi\zeta_1 + \psi\zeta_2)^2 \rangle \\
&= \beta^2 \left[2\phi\psi\langle\zeta_1\zeta_2\rangle + \psi^2\langle\zeta_2^2\rangle + \phi^2\langle\zeta_1^2\rangle \right] \\
&= \beta^2 \left[2\phi\psi\Theta_{12} + \psi^2\Theta_{22} + \phi^2\Theta_{11} \right]
\end{aligned}
$$

1.2.2.2 lag-τ autocorrelation

To derive an analytical approximation for autocorrelation of the rate of incidence (RoI, η), we take the Fourier Transformation of the identity, $\eta = \beta(\phi\zeta_1 + \psi\zeta_2)$. From linearity of the Fourier Transform (as considering this identity evaluated at fixed points, ϕ^* and ψ^2),

$$\tilde{\eta} = \beta(\phi\tilde{\zeta_1} + \psi\tilde{\zeta_2})$$

For this reason, we also require the FT of the susceptible fluctuations ζ_1, which we can similarly derive along with the Power Spectrum to be,

$$\tilde{\zeta_1} = \frac{1}{d - it\omega - \omega^2}(a_{12}\Gamma_2 + (a_{22} - i\omega)\Gamma_1)$$
$$S_{\zeta_1}(\omega) = \frac{a_{22}^2 B_{11} - 2a_{12}a_{22}B_{12} + a_{12}^2 B_{22} + B_{11}\omega^2}{(d - \omega^2)^2 + t^2\omega^2}$$

Bringing this together, we achieve,

$$\tilde{\eta} = \frac{\beta}{d - it\omega - \omega^2}\left[(\psi a_{21} + \phi(a_{22} - i\omega))\Gamma_1 + (\phi a_{12} - \psi(a_{11} - i\omega))\Gamma_2\right]$$
$$\begin{aligned} S_\eta(\omega) = &\frac{\beta^2}{(d - \omega^2)^2 + t^2\omega^2}((a_{22}^2\phi^2 - 2a_{21}a_{22}\phi\psi + a_{21}^2\psi^2)B_{11} \\ &+ (a_{12}^2\phi^2 - 2a_{12}a_{11}\phi\psi + \psi^2 a_{11}^2)B_{22} \\ &+ 2((a_{12}a_{21} + a_{22}a_{11})\phi\psi - a_{22}a_{12}\phi^2 - a_{21}a_{11}\psi^2)B_{12} \\ &+ (\phi^2 B_{11} + \psi^2 B_{22} + 2\phi\psi B_{12})\omega^2) \end{aligned}$$

1.3 SIS Emergence

1.3.1 Prevalence

This model includes external force of infection, governed by parameter ν, making it an extension of the SIS model shown in section 1.1. The following work has been derived by O'Regan and Drake (3) and has been reproduced for ease of the reader to make comparisons to other models.

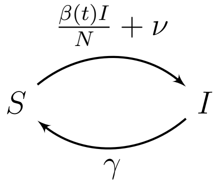

As such, only the transition rate $T(I+I|I)$ changes and becomes $\frac{\beta(t)(N-I)I}{N}+\nu(N-I)$. Hence,

$$\frac{dP(I,t)}{dt}=(\mathbb{E}^{-1}-1)(\frac{\beta(t)(N-I)I}{N}+\nu(N-I))P(I,t)+(\mathbb{E}-1)\gamma IP(I,t)$$

Following this new transition through, we achieve a similar FPE:

$$\begin{aligned}\frac{\partial\Pi}{\partial t} &= N^{1/2}(\beta\phi(1-\phi)-\gamma\phi+\nu(1-\phi))\frac{\partial\Pi}{\partial\zeta}+(N^{1/2}\frac{\partial}{\partial\zeta}+\frac{1}{2}\frac{\partial^2}{\partial\zeta^2})\gamma(\phi+N^{-1/2}\zeta)\Pi(\zeta,t)\\ &+ (-N^{1/2}\frac{\partial}{\partial\zeta}+\frac{1}{2}\frac{\partial^2}{\partial\zeta^2})(\beta\phi(1-\phi)+\beta(1-2\phi)N^{-1/2}\zeta-\beta N^{-1}\zeta^2)\Pi(\zeta,t)\\ &+ (-N^{1/2}\frac{\partial}{\partial\zeta}+\frac{1}{2}\frac{\partial^2}{\partial\zeta^2})\nu(1-\phi-N^{-1/2}\zeta)\Pi(\zeta,t)\\ \frac{\partial\Pi}{\partial t} &= -(\beta(1-2\phi)-\gamma-\nu)\frac{\partial\zeta\Pi}{\partial\zeta}+\frac{1}{2}(\beta(1-\phi)\phi+\gamma\phi+\nu(1-\phi))\frac{\partial^2\Pi}{\partial\zeta^2}.\end{aligned}$$

1.3.1.1 Variance

Using the FPE, we can multiple through by ζ^2 and integrate this over the domain to achieve the variance of the fluctuation,

$$\frac{\partial\langle\zeta^2\rangle_t}{\partial t}=2(\beta(1-2\phi)-\gamma-\nu)\langle\zeta^2\rangle_t+\beta(1-\phi)\phi+\gamma\phi+\nu(1-\phi) \tag{30}$$

1.3.1.2 lag-τ autocorrelation

We again extend the work from SIS model with social distancing using a Fourier Transformation of ζ about the quasi stationary state. To this extent we achieve,

$$\begin{aligned}\tilde{\zeta} &= \frac{Q^{1/2}\tilde{\gamma}}{i\omega-\lambda}\\ \lambda &= (\beta(1-2\phi)-\gamma-\nu)\\ Q &= \beta(1-\phi)\phi+\gamma\phi+\nu(1-\phi)\end{aligned}$$

Then the power spectrum, $S(\omega)$ is,

$$S(\omega)=\frac{Q}{\omega^2+\lambda^2}$$

And the variance and autocorrelation are,

$$\sigma^2 = \frac{Q}{2|\lambda|}$$
$$ACF = e^{-|\lambda|}$$
$$= e^{-|\beta(1-2\phi)-\gamma-\nu|}$$

1.3.2 Rate of Incidence

We define the rate of new infectious cases to be given by the incoming transition probability in class I,

$$n_c = T(I|I-1) = \beta SI + \nu S$$

.

We are interested in the fluctuations about this rate, as such we similarly consider the LNA:

$$n_c = N\sigma + N^{1/2}\omega$$
$$\sigma = \beta\phi(1-\phi) + \nu(1-\phi)$$

n_c can also be written in terms of the fluctuations about I (defined by variable ζ above),

$$n_c = \beta SI/N + \nu S$$
$$= N\beta\phi(1-\phi) + N\nu(1-\phi) + N^(1/2)(\beta(1-2\phi)-\nu)\zeta - -\beta\zeta^2$$
$$\eta = (\beta(1-2\phi)-\nu)\zeta - \beta N^{-1/2}\zeta^2$$
$$\text{Take the limit } N \to \infty$$
$$\eta = (\beta(1-2\phi(t))-\nu)\zeta = f(\zeta, t)$$

We can use the previously derived results for the SDE describing $d\zeta = (\beta(1-2\phi)-\gamma-\nu)dt + \sqrt{\beta(1-\phi)\phi+\gamma\phi+\nu(1-\phi)}dW_t$ with Ito's change of variable formula for a function dependent on t: $f(\zeta, t)$, to evaluate:

$$df(\zeta, t) = f_\zeta d\zeta + f_t dt + 1/2 f_{\zeta\zeta} b^2 dt$$
$$df(\zeta, t) = \left[(\beta(1-2\phi)-\nu)(\beta(1-2\phi)-\gamma-\nu)\zeta - 2\beta\zeta(\beta\phi(1-\phi)-\gamma\phi+\nu(1-\phi))\right] dt$$
$$+ (\beta(1-2\phi)-\nu)\sqrt{\beta\phi(1-\phi)+\gamma\phi+\nu(1-\phi)}dW_t$$
$$d\eta = \left[(\beta(1-2\phi)-\gamma-\nu) - \frac{2\beta}{\beta(1-2\phi)-\nu}(\beta\phi(1-\phi)-\gamma\phi+\nu(1-\phi))\right]\eta dt$$
$$+ (\beta(1-2\phi)-\nu)\sqrt{\beta\phi(1-\phi)+\gamma\phi+\nu(1-\phi)}dW_t$$

The Fokker-Planck equation which is equivalent to the above SDE is given by,

$$\frac{d\Pi(\eta,t)}{dt} = -\frac{\partial}{\partial\eta}\left((\beta(1-2\phi)-\gamma-\nu) - \frac{2\beta}{\beta(1-2\phi)-\nu}(\beta\phi(1-\phi)-\gamma\phi+\nu(1-\phi))\right)\eta\Pi(\eta,t)$$
$$+ \frac{1}{2}\frac{\partial^2}{\partial\eta^2}\big((\beta(1-2\phi)-\nu)^2(\beta\phi(1-\phi)+\gamma\phi+\nu(1-\phi))\Pi(\eta,t)\big)$$

1.3.2.1 Variance

The variance of η is given by,

$$\frac{d<\eta^2>}{dt} = 2\left((\beta(1-2\phi)-\gamma-\nu) - \frac{2\beta}{\beta(1-2\phi)-\nu}(\beta\phi(1-\phi)-\gamma\phi-\nu(1-\phi))\right) <\eta^2>$$
$$+ (\beta(1-2\phi)-\nu)^2(\beta\phi(1-\phi)+\gamma\phi+\nu(1-\phi))$$

1.3.2.2 lag-τ autocorrelation

It can be shown similarly that the Fourier Transform of η.

As such, we find that

$$\tilde{\eta} = \frac{Q^{1/2}\tilde{\gamma}}{i\omega - \lambda}$$
$$\lambda = (\beta(1-2\phi) - \gamma - \nu) - \frac{2\beta}{\beta(1-2\phi)-\nu}(\beta\phi(1-\phi) - \gamma\phi + \nu(1-\phi))$$
$$Q = (\beta(1-2\phi) - \nu)^2(\beta\phi(1-\phi) + \gamma\phi + \nu)$$

Then the lag-1 ACF is $e^{-|\lambda|} = e^{-|\beta(1-2\phi)-\gamma-\nu)-\frac{2\beta}{\beta(1-2\phi)-\nu}(\beta\phi(1-\phi)-\gamma\phi+\nu(1-\phi))|}$.

1.3.3 Incidence results from O'Dea *et al.*

Results by O'Dea *et al.* finding the mean ($\langle N(\Delta t)\rangle$) and variance ($var(N(\Delta t))$) of incidence-type data evaluated at steady state for the BDI process are given,

$$\langle N(\Delta t)\rangle = \frac{\nu\gamma\Delta t}{\gamma - \beta(\Delta t)},$$
$$\omega = \frac{(\gamma - \beta(\Delta t))\Delta t}{2}$$
$$N(\Delta t)^{[2]} = 1 + \frac{\beta(\Delta t)}{\nu\omega}\left(1 - \frac{1 - exp(-2\omega)}{2\omega}\right),$$
$$var(N(\Delta t)) = \langle N(\Delta t)\rangle^2(N(\Delta t)^{[2]} - 1) + \langle N(\Delta t)\rangle$$

where $N(\Delta t)$ is the number of infectious individuals who are removed in the period Δt.

References

[1] Van Kampen NG. Stochastic processes in physics and chemistry. Elsevier; 1992 Nov 20.

[2] Dessavre AG, Southall E, Tildesley MJ, Dyson L. The problem of detrending when analysing potential indicators of disease elimination. Journal of theoretical biology. 2019 Apr 11.

[3] O'Regan SM, Drake JM. Theory of early warning signals of disease emergenceand leading indicators of elimination. Theoretical Ecology. 2013 Aug 1;6(3):333-57.
