# Supplementary material for: Prospects for detecting early warning signals in discrete event sequence data: Application to epidemiological incidence data
Source: PLoS Comput Biol. 2020 Sep 22;16(9):e1007836. doi: 10.1371/journal.pcbi.1007836 (PMC7531856; doi:10.1371/journal.pcbi.1007836)
Supplement: S1 Table — (PDF) [file pcbi.1007836.s002.pdf]

Table of Indicators

To calculate the statistic at each timepoint of our simulations X, we first detrend by removing the mean over $R = 500$ realisations, we name this detrend simulation by x. We then calculate the statistic over the detrended realisations,

$$\hat{X}(t) = \frac{1}{R}\sum_{i}^{R} X_i(t)$$
$$x(t) = X(t) - \hat{X}(t)$$

EWS	Simulation Formula	Theoretical Prediction
Variance (V)	$\sigma^2(t) = \frac{1}{R}\sum_{i=1}^{R} x_i(t)^2$	Section: 1.1, 1.2, 1.3 (Prev, RoI); $V = \Delta t\lambda(t) = \Delta t T(I+1\|I)$ (Inc)
Coefficient of Variation (CoV)	$CV(t) = \frac{\sigma(t)}{\hat{X}(t)}$	Section: 1.1, 1.2, 1.3 (Prev, RoI); $CV = \frac{1}{\sqrt{\Delta t\lambda(t)}} = \frac{1}{\sqrt{\Delta t T(I+1\|I)}}$ (Inc)
Skewness	$SK(t) = \frac{\frac{1}{R}\sum_{i=1}^{R} x_i(t)^3}{\sigma^3}$	Zero (Prev, RoI), $SK(t) = \frac{1}{\sqrt{\Delta t\lambda(t)}} = \frac{1}{\sqrt{\Delta t T(I+1\|I)}}$
Excess Kurtosis	$KT(t) = \frac{\frac{1}{R}\sum_{i=1}^{R} x_i(t)^4}{\sigma^4} - 3$	Section: 1.1, 1.2, 1.3 (Prev, RoI); $KT(t) = \frac{1}{\Delta t\lambda(t)} = \frac{1}{\Delta t T(I+1\|I)}$ (Inc)
Autocorrelation lag-1	$AC_i(1) = \frac{1}{W}\sum_{t'=t-W/2}^{t+W/2}(x_i(t') - \hat{x_W}(t'))(x_i(t'+1) - \hat{x_W}(t'))$ $AC(1) = \frac{1}{R}\sum_{i=1}^{R} AC_i(1)$	Section: 1.1, 1.2, 1.3 (Prev, RoI)

Table S1. List of early warning signals.
