# Supplementary material for: Prospects for detecting early warning signals in discrete event sequence data: Application to epidemiological incidence data
Source: PLoS Comput Biol. 2020 Sep 22;16(9):e1007836. doi: 10.1371/journal.pcbi.1007836 (PMC7531856; doi:10.1371/journal.pcbi.1007836)
Supplement: S1 Fig — For each model (Model 1 (panel a and b): SIS with social distancing (elimination); Model 2 (panel c and d): SIS increasing vaccination (elimination); Model 3 (panel e and f): SIS increasing transmission (emergence) we calculate the variance between 500 homogeneous realisations subject to aggregation of time steps (Δt). We take the average of new cases over time step aggregation. Each figure shows: theory (dotted black line); critical transition (dashed black line) and Gillespie simulations (colours correspond to time aggregation). The last model also shows the dynamical prediction from O’Dea which was derived for this specific system (solid black line). (PDF) [file pcbi.1007836.s003.pdf]

Sensitivity to time aggregation (Δt) when approximating non-homogen
Poisson process

For the parameters we have chosen (Table 1, main text) we find that approximating the Poisson process rate by,

$$\lambda(t) = \int_t^{t+\Delta t} T(I(t)+1|I(t))dt \approx \Delta t T(I(t)+1|I(t)),$$

holds for up to $\Delta t = 3$ when considering the statistic variance and for other indicators holds for $\Delta t > 7$ (not shown here). This is subject to parameters chosen for simulation run times and therefore for future studies should explore the rate of approach of the critical transition and quantity of data points.

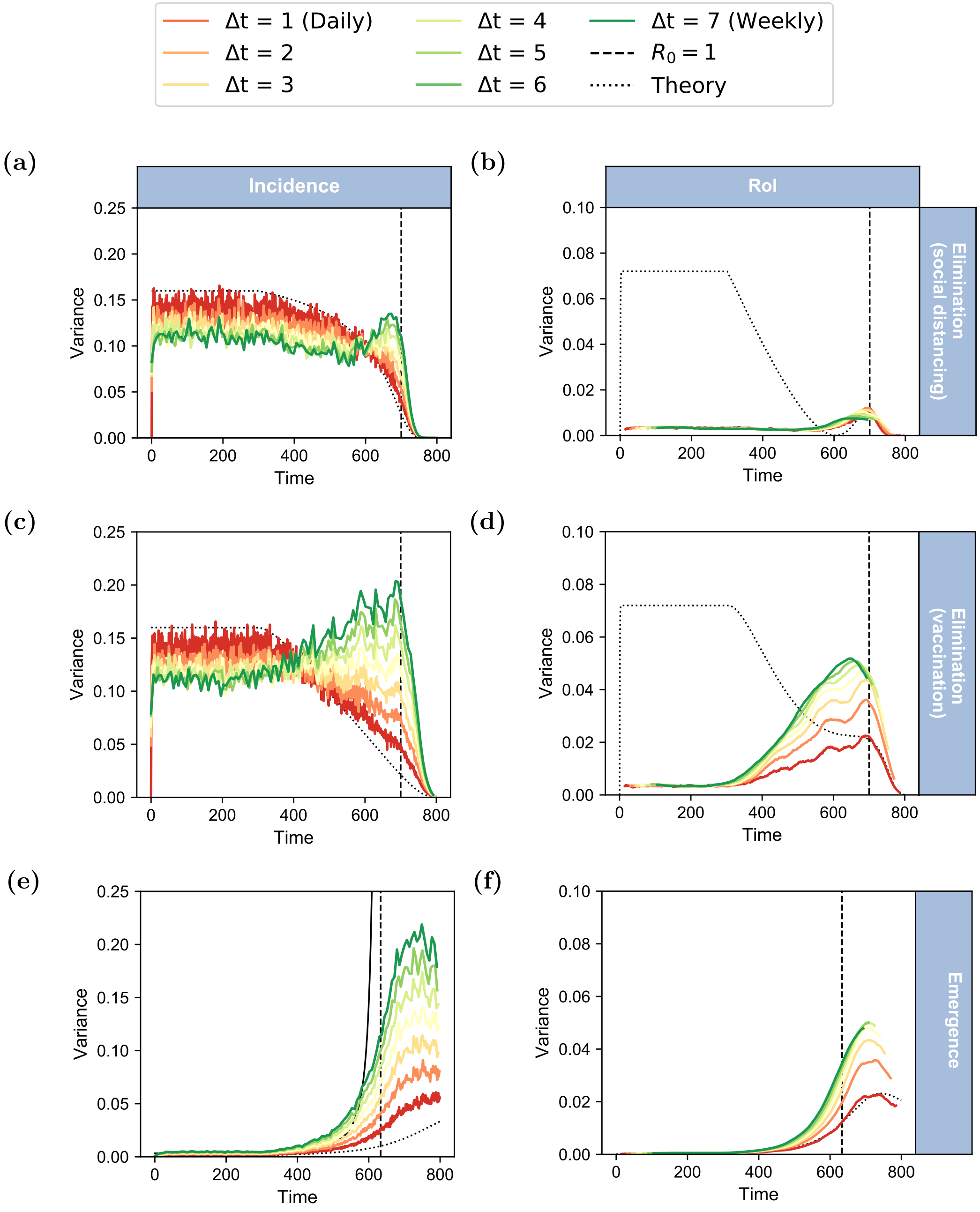

Fig. S1. Sensitivity to time aggregation (Δt) when approximating non-homogeneous Poisson process. For each model (Model 1 (panel a and b): SIS with social distancing (elimination); Model 2 (panel c and d): SIS increasing vaccination (elimination); Model 3 (panel e and f): SIS increasing transmission (emergence) we calculate the variance between 500 homogeneous realisations subject to aggregation of time steps (Δt). We take the average of new cases over time step aggregation. Each figure shows: theory (dotted black line); critical transition (dashed black line) and Gillespie simulations (colours correspond to time aggregation). The last model also shows the dynamical prediction from O'Dea which was derived for this specific system (solid black line).
