# Supplementary material for: Prospects for detecting early warning signals in discrete event sequence data: Application to epidemiological incidence data
Source: PLoS Comput Biol. 2020 Sep 22;16(9):e1007836. doi: 10.1371/journal.pcbi.1007836 (PMC7531856; doi:10.1371/journal.pcbi.1007836)
Supplement: S2 Fig — For each model: SIS with social distancing (a), SIS with vaccination (b), SIS increasing transmission/emergence (c) and the SIR epidemic curve (d) we plot two approximations of the intensity of a Poisson process. The dashed line is the point where R0 = 1 or Reff = 1 (effective reproduction number—SIR model). Blue line is variance calculated over stochastic simulations. Orange line: Poisson Process with λ(t) = γI(t). Green line: Poisson Process with λ(t) = βS(t)I(t). Panel (d): SIR epidemic curve, is included here to demonstrate an example where the approximation βSI/N ≈ γI isn’t appropriate. This is the SIR model with: β(t) = 0.1 and γ = 0.01. (PDF) [file pcbi.1007836.s004.pdf]

Approximating incidence by using the rate of the Poisson Process, $\lambda(t) = \Delta t \gamma I$

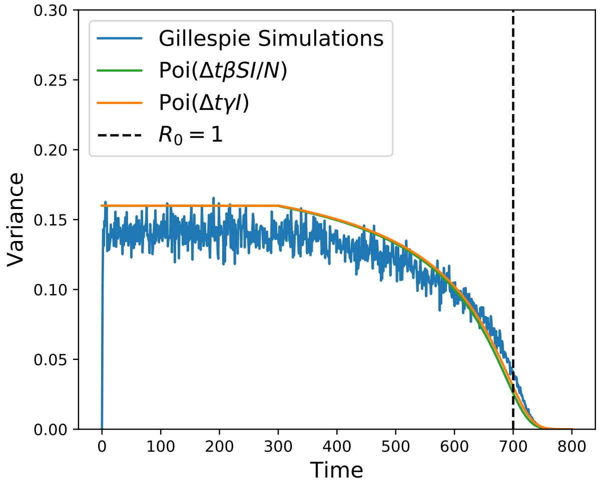

(a) SIS - social distancing (elimination)

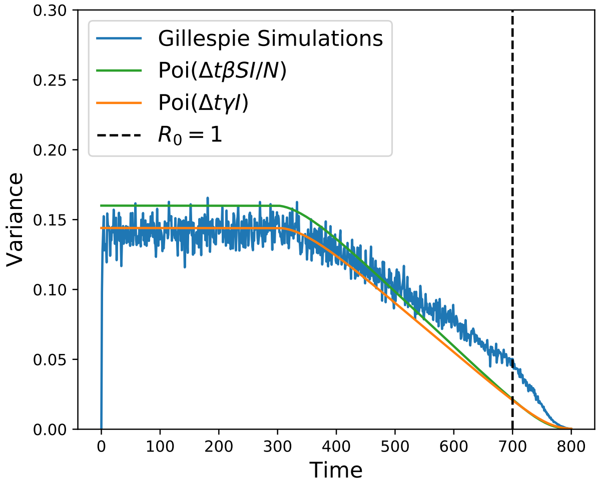

(b) SIS - vaccination (elimination)

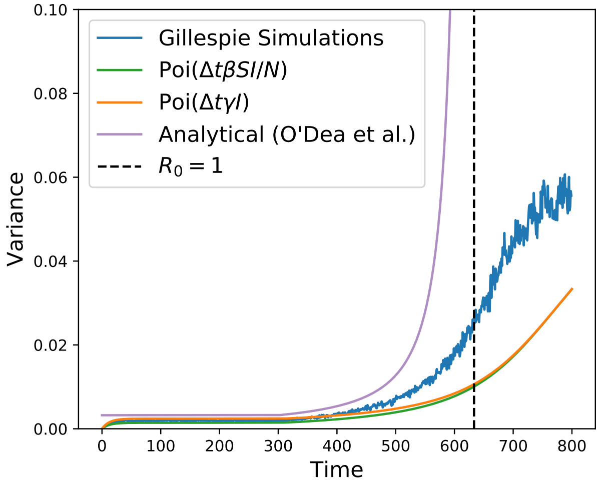

(c) SIS (emergence)

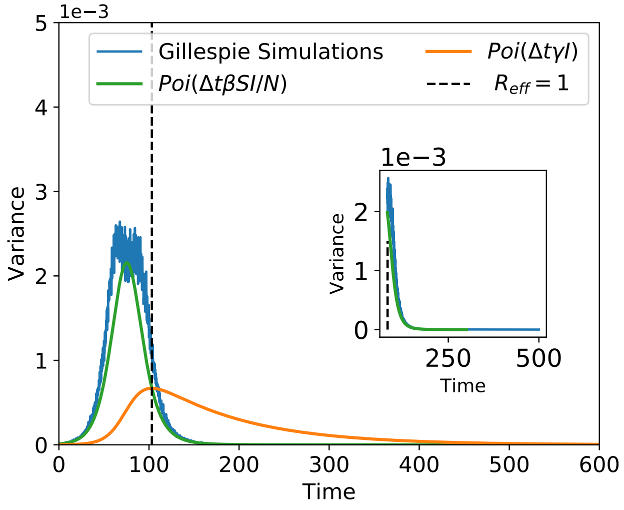

(d) SIR: epidemic curve (emergence and elimination phase)

Fig. S2. Approximating incidence by using the rate of the Poisson Process, $\lambda(t) = \Delta t \gamma I$. For each model: SIS with social distancing (a), SIS with vaccination (b), SIS increasing transmission/emergence (c) and the SIR epidemic curve (d) we plot two different approximations of the intensity of a Poisson process. The dashed line is the point where $R_0 = 1$ or $R_{eff} = 1$ (effective reproduction number - SIR model). Blue line is variance calculated over stochastic simulations. Orange line: Poisson Process with $\lambda(t) = \gamma I(t)$. Green line: Poisson Process with $\lambda(t) = \beta S(t) I(t)$. Panel (d): SIR epidemic curve, is included here to demonstrate an example where the approximation $\beta SI/N \approx \gamma I$ isn't appropriate. This is the SIR model with: $\beta(t) = 0.1$ and $\gamma = 0.01$
