# Supplementary material for: Prospects for detecting early warning signals in discrete event sequence data: Application to epidemiological incidence data
Source: PLoS Comput Biol. 2020 Sep 22;16(9):e1007836. doi: 10.1371/journal.pcbi.1007836 (PMC7531856; doi:10.1371/journal.pcbi.1007836)
Supplement: S3 Fig — SIS with social distancing (elimination) and parameters β0 = 0.24. In this example, the dynamics follow those of Section 1.1 but with different parameters. This leads to the approach towards R0 = 1 being at a slower rate where R0 changes from 1.2 to 0. (PDF) [file pcbi.1007836.s005.pdf]

Slower rate reduces discrepancies between theory and simulated variance in “rolling” RoI

We observe that the “true” simulated data of the rate of new cases (calculated as the product βSI, purple line) can be predicted well by the theoretical solution (derived above, orange line) for all models.

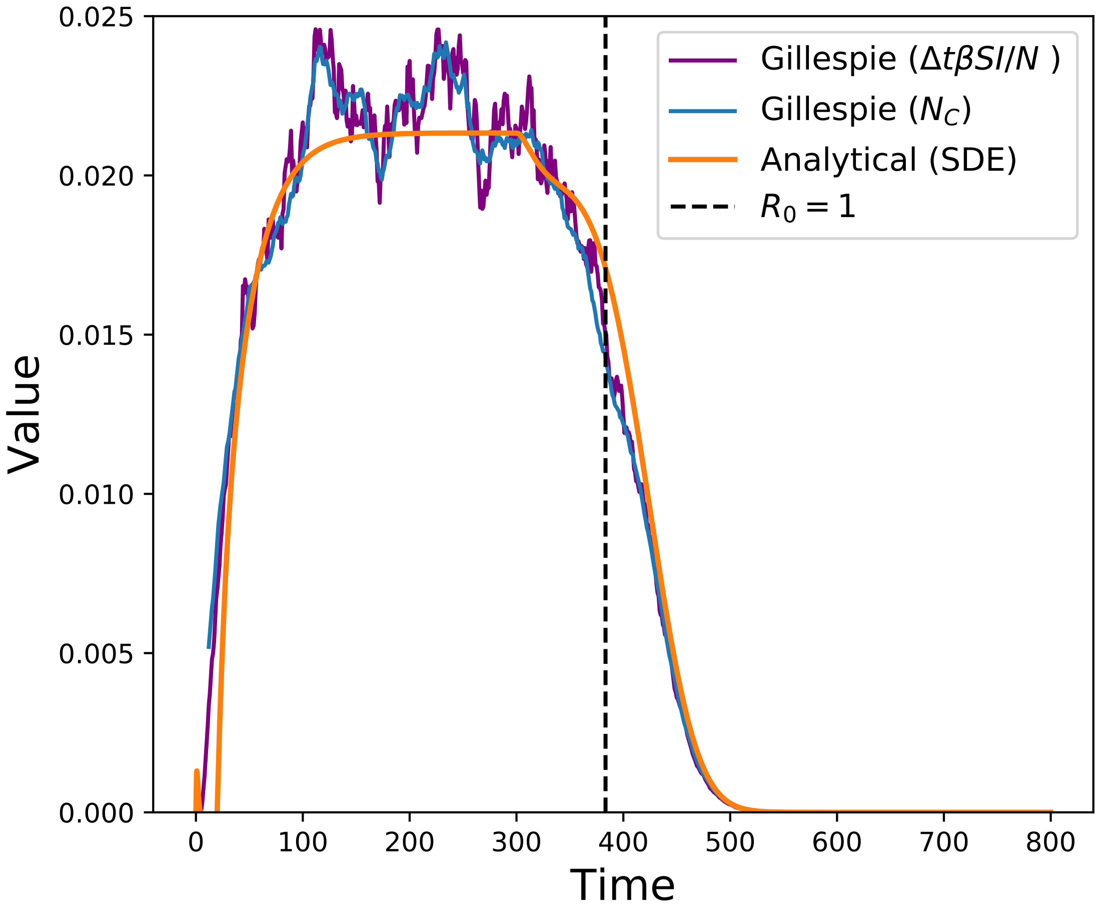

Fig. S3. Slower rate reduces discrepancies between theory and simulated variance in “rolling” RoI. SIS with social distancing (elimination) and parameters $\beta_0 = 0.24$. In this example, the dynamics follow those of Section 1.1 but with different parameters. This leads to the approach towards $R_0 = 1$ being at a slower rate where R_0 changes from 1.2 to 0.

However the approximated solution of the rate of new cases (moving average of incidence, blue line) can be over dispersed, particularly for elimination models. We demonstrate in Figure SS3, that as we reduce the rate of approach towards the critical transition the approximated solution (blue line) converges to the theory. This can explain why we observe better performance for SIS emergence for the approximated solution, when compared to SIS with social distancing and with vaccination.
