# Supplementary material for: Prospects for detecting early warning signals in discrete event sequence data: Application to epidemiological incidence data
Source: PLoS Comput Biol. 2020 Sep 22;16(9):e1007836. doi: 10.1371/journal.pcbi.1007836 (PMC7531856; doi:10.1371/journal.pcbi.1007836)
Supplement: S4 Fig — Calculating “rolling” RoI with a range of bandwidths for: a). SIS with social distancing, b). SIS with vaccination and c). SIS emergence. (PDF) [file pcbi.1007836.s006.pdf]

Approximation method “rolling” RoI is consistent across different bandwidth sizes

We observe that the “true” simulated data of the rate of new cases (calculated as the product βSI, purple line) can be predicted well by the theoretical solution (derived above, orange line) for all models.

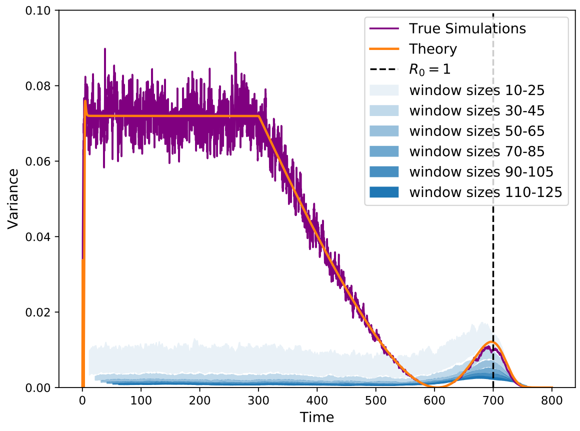

(a) SIS - social distancing (elimination)

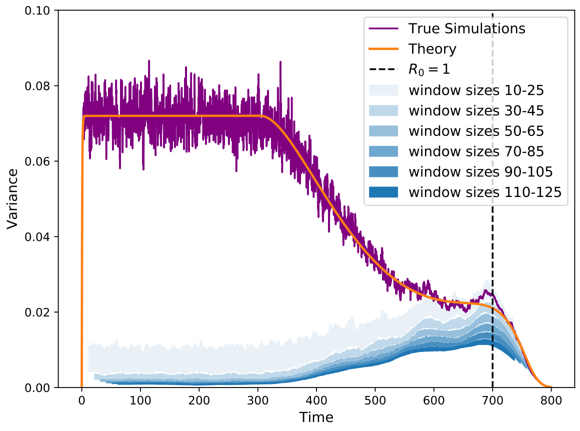

(b) SIS - vaccination (elimination)

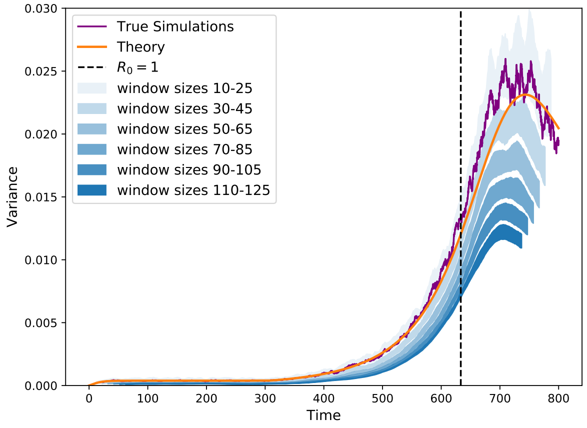

(c) SIS (emergence)

Fig. S4. Approximation method “rolling” RoI is consistent across different bandwidth sizes. Calculating “rolling” RoI with a range of bandwidths for: a). SIS with social distancing, b). SIS with vaccination and c). SIS emergence.

However the approximated solution of the rate of new cases (moving average of incidence, blue line) can be under dispersed, particularly for elimination models. We demonstrate in Figure S S4, that for a large range of bandwidth sizes (width of moving window), the timeseries trend of “rolling” RoI is robust. We tested bandwidth sizes between $[10, 125]$ in steps of 5, we observe from Figure SS4 that the increased peak as the threshold is approached is pronounced and exhibited for all sizes.
