# Supplementary material for: Prospects for detecting early warning signals in discrete event sequence data: Application to epidemiological incidence data
Source: PLoS Comput Biol. 2020 Sep 22;16(9):e1007836. doi: 10.1371/journal.pcbi.1007836 (PMC7531856; doi:10.1371/journal.pcbi.1007836)
Supplement: S5 Fig — For each model: a,b,c).SIS social distancing (elimination); d,e,f). SIS increasing vaccination (elimination); g, h, i). SIS increasing transmission, (emergence) we calculate the variance between 500 homogeneous realisations at every time step (daily). Each figure shows: Poisson Process distribution (green line); dynamic predictions (red line) and Gillespie simulations (Ext and Emg, blue line). The last model also shows the dynamical prediction from O’Dea which was derived for this specific system (orange line). (PDF) [file pcbi.1007836.s007.pdf]

Variance

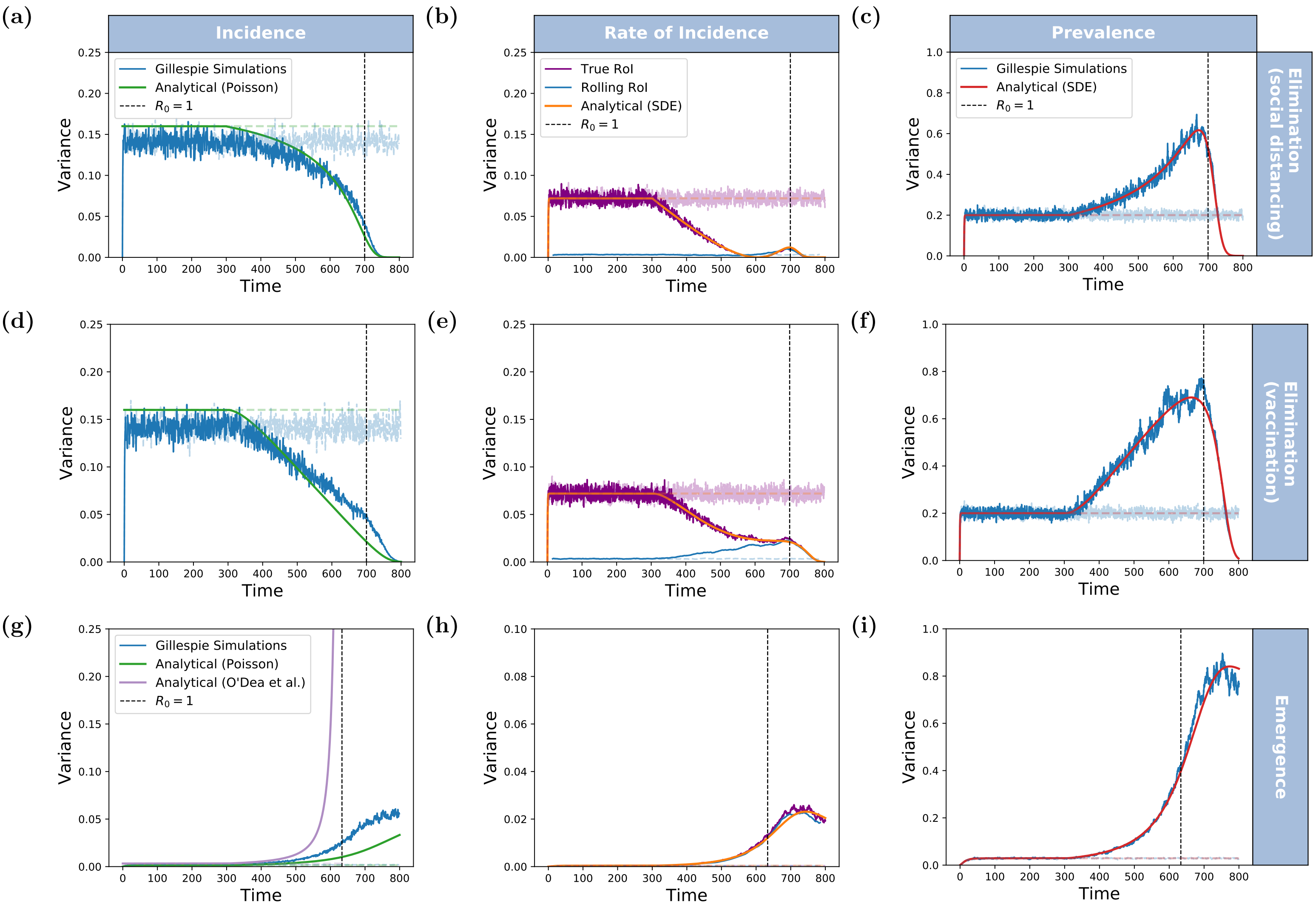

Fig. S5. Comparing predictions to simulations for Variance For each model: a,b,c).SIS social distancing (elimination); d,e,f). SIS increasing vaccination (elimination); g, h, i). SIS increasing transmission, (emergence) we calculate the variance between 500 homogeneous realisations at every time step (daily). Each figure shows: Poisson Process distribution (green line); dynamic predictions (red line) and Gillespie simulations (Ext and Emg, blue line). The last model also shows the dynamical prediction from O'Dea which was derived for this specific system (orange line).
