# Supplementary material for: Prospects for detecting early warning signals in discrete event sequence data: Application to epidemiological incidence data
Source: PLoS Comput Biol. 2020 Sep 22;16(9):e1007836. doi: 10.1371/journal.pcbi.1007836 (PMC7531856; doi:10.1371/journal.pcbi.1007836)
Supplement: S6 Fig — For each model: a, b, c). SIS social distancing (elimination); d,e,f). SIS increasing vaccination (elimination); g,h,i). SIS increasing transmission, (emergence) we calculate the CV between 500 homogeneous realisations at every time step (daily). Each figure shows: Poisson Process distribution (green line); dynamic predictions (red line) and Gillespie simulations (Ext and Emg, blue line). (PDF) [file pcbi.1007836.s008.pdf]

Coefficient of Variation

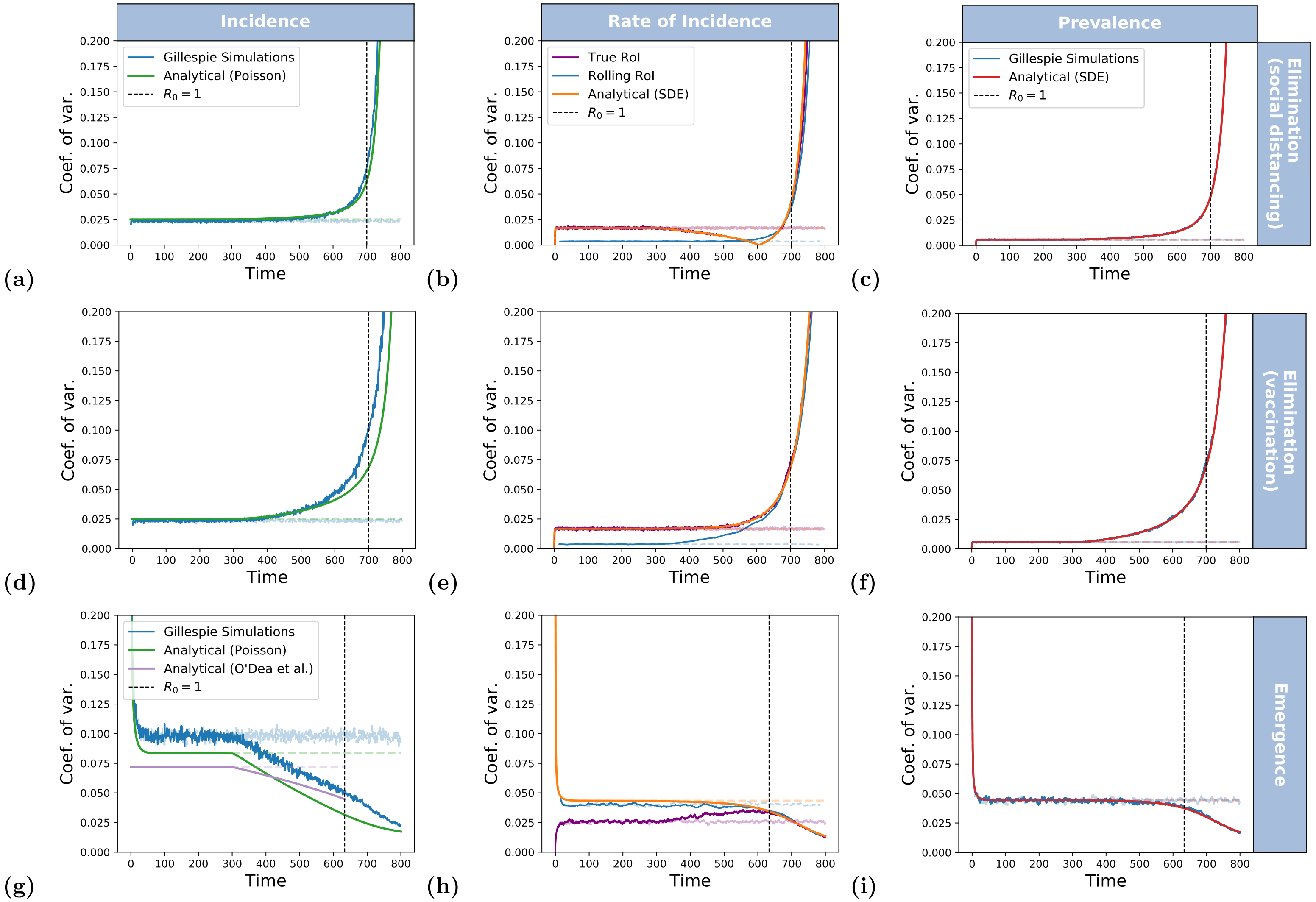

Fig. S6. Comparing predictions to simulations for Coefficient of Variation For each model: a, b, c). SIS social distancing (elimination); d,e,f). SIS increasing vaccination (elimination); g,h,i). SIS increasing transmission, (emergence) we calculate the CV between 500 homogeneous realisations at every time step (daily). Each figure shows: Poisson Process distribution (green line); dynamic predictions (red line) and Gillespie simulations (Ext and Emg, blue line).
