# Supplementary material for: Prospects for detecting early warning signals in discrete event sequence data: Application to epidemiological incidence data
Source: PLoS Comput Biol. 2020 Sep 22;16(9):e1007836. doi: 10.1371/journal.pcbi.1007836 (PMC7531856; doi:10.1371/journal.pcbi.1007836)
Supplement: S9 Fig — S9A: Comparing predictions to simulations for autocorrelation lag-1 For each model: a,b,c). SIS social distancing (elimination); d,e,f). SIS increasing vaccination (elimination); g,h,i). SIS increasing transmission, (emergence) at each point t we calculate the lag-1 AC on an interval [t − W/2, t+ W/2]. We then calculate the mean of this AC over 500 homogeneous realisations at every time step. Each figure shows: Poisson Process distribution (green line); dynamic predictions (red line) and Gillespie simulations (Ext and Emg, blue line). S9B: Evaluating the MSE between theoretical prediction and simulations for different window sizes. For each mode, we calculate how the error between theoretical solution and simulations changes with different window sizes used in the moving average calculated of AC(1). We demonstrate that these errors narrow, and define the minimum window size of which the error is below some threshold (given in the plots) to be chosen suitable window size (for the moving average calculations). (PDF) [file pcbi.1007836.s011.pdf]

Autocorrelation

Let W be the window size of the moving average and τ be the lag of the AC,

$$AC_\tau(t)_i = \frac{1}{W}\sum_{t'=t-W/2}^{t+W/2} x_i(t')x_i(t'+\tau)$$

$$AC_\tau(t) = \frac{1}{R}\sum_{i=1}^{R} AC_\tau(t)_i$$

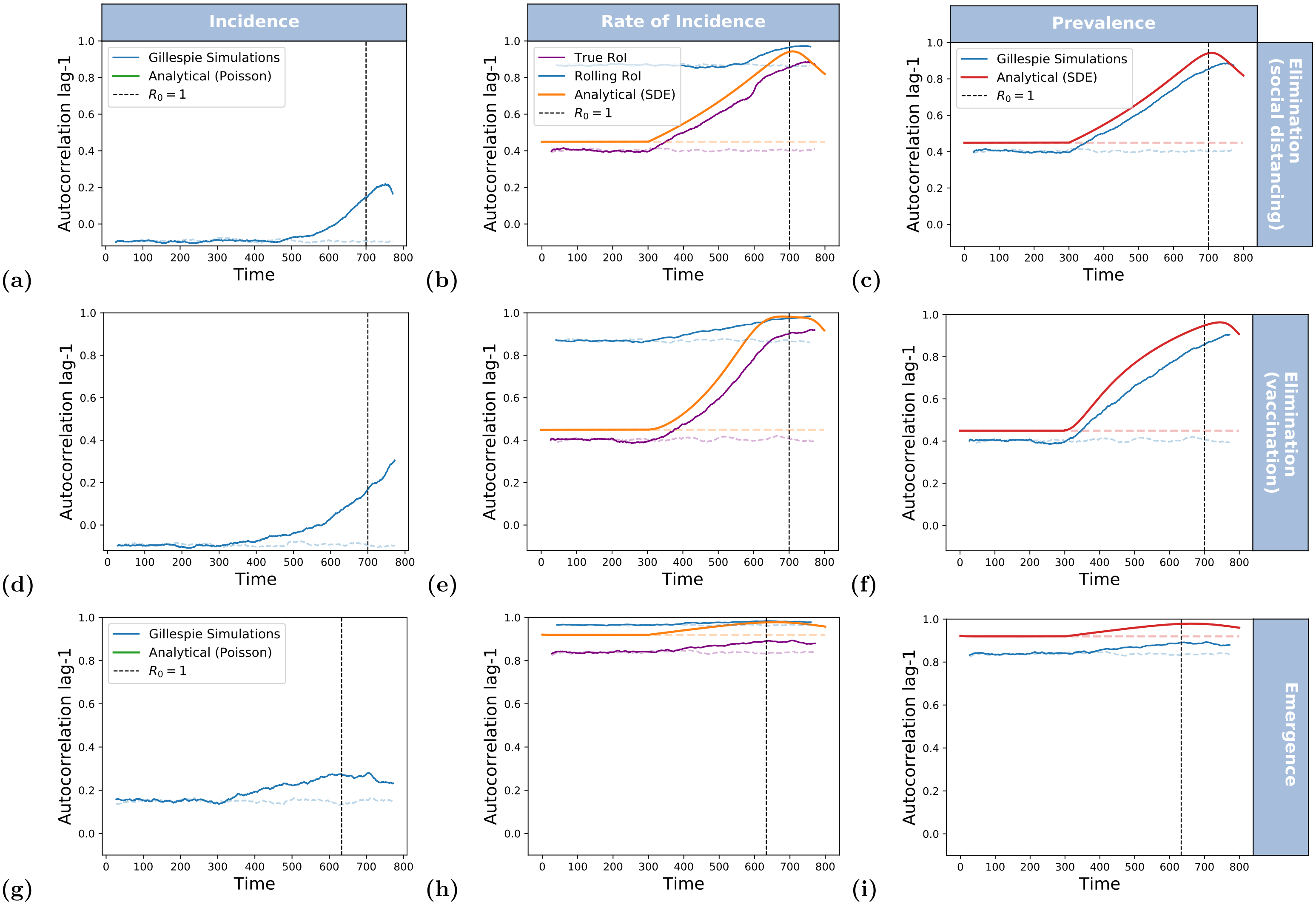

Fig. S9A. Comparing predictions to simulations for autocorrelation lag-1 For each model: a,b,c). SIS social distancing (elimination); d,e,f). SIS increasing vaccination (elimination); g,h,i). SIS increasing transmission, (emergence) at each point t we calculate the lag-1 AC on an interval $[t - W/2, t + W/2]$. We then calculate the mean of this AC over 500 homogeneous realisations at every time step. Each figure shows: Poisson Process distribution (green line); dynamic predictions (red line) and Gillespie simulations (Ext and Emg, blue line).

Choosing a suitable window size

We show that are the moving window size is increased (increasing the interval size of the calculation of AC lag-1), the simulated solutions get closer to the quasi-stationary theory of ACF.

We demonstrate this by measuring the mean squared error between the simulated results of ACF and the corresponding theoretical solution. Below, we present how these curve converge as the window size is increased.

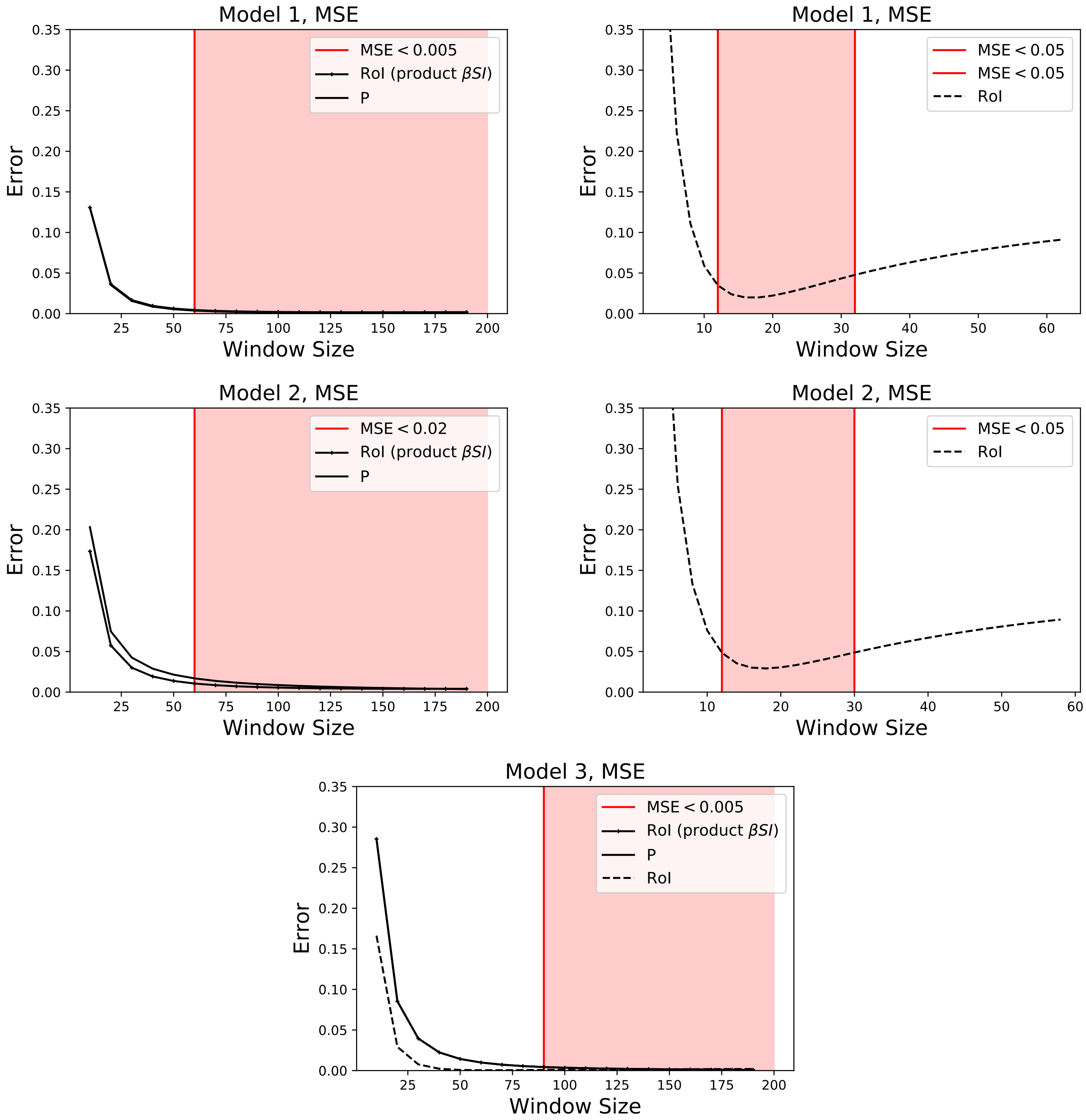

Fig. S9B. Evaluating the MSE between theoretical prediction and simulations for different window sizes. For each mode, we calculate how the error between theoretical solution and simulations changes with different window sizes used in the moving average calculated of AC(1). We demonstrate that these errors narrow, and define the minimum window size of which the error is below some threshold (given in the plots) to be chosen suitable window size (for the moving average calculations).
