# Supplementary material for: Prospects for detecting early warning signals in discrete event sequence data: Application to epidemiological incidence data
Source: PLoS Comput Biol. 2020 Sep 22;16(9):e1007836. doi: 10.1371/journal.pcbi.1007836 (PMC7531856; doi:10.1371/journal.pcbi.1007836)
Supplement: S10 Fig — Comparison of disease transitioning simulations: SIS with social distancing (solid lines); SIS with vaccination (dashed lines) and SIS disease emergence with increasing transmission (dotted lines) to the null model (Fixed endemic simulations). Each curve compares the null simulations to each model for different indicators and for each data type: prevalence (P, red lines), incidence (I, green lines) and rate of incidence (RoI, orange lines). ROC curves calculated for indicators: variance; coefficient of variation (CV) and lag-1 autocorrelation (AC). Kendall-tau scores measured over each data-type up to t1 = 390 (pre disease transmission) are given in the left column and up to t2 = 450 (post disease transmission) are given in the right column. For each ROC curve we measured the AUC score which is given in legend, which is an indication of how predictive each indicator is by its ability to distinguish between elimination simulations and the null model. (PDF) [file pcbi.1007836.s012.pdf]

Receiver Operator Characteristic Curves

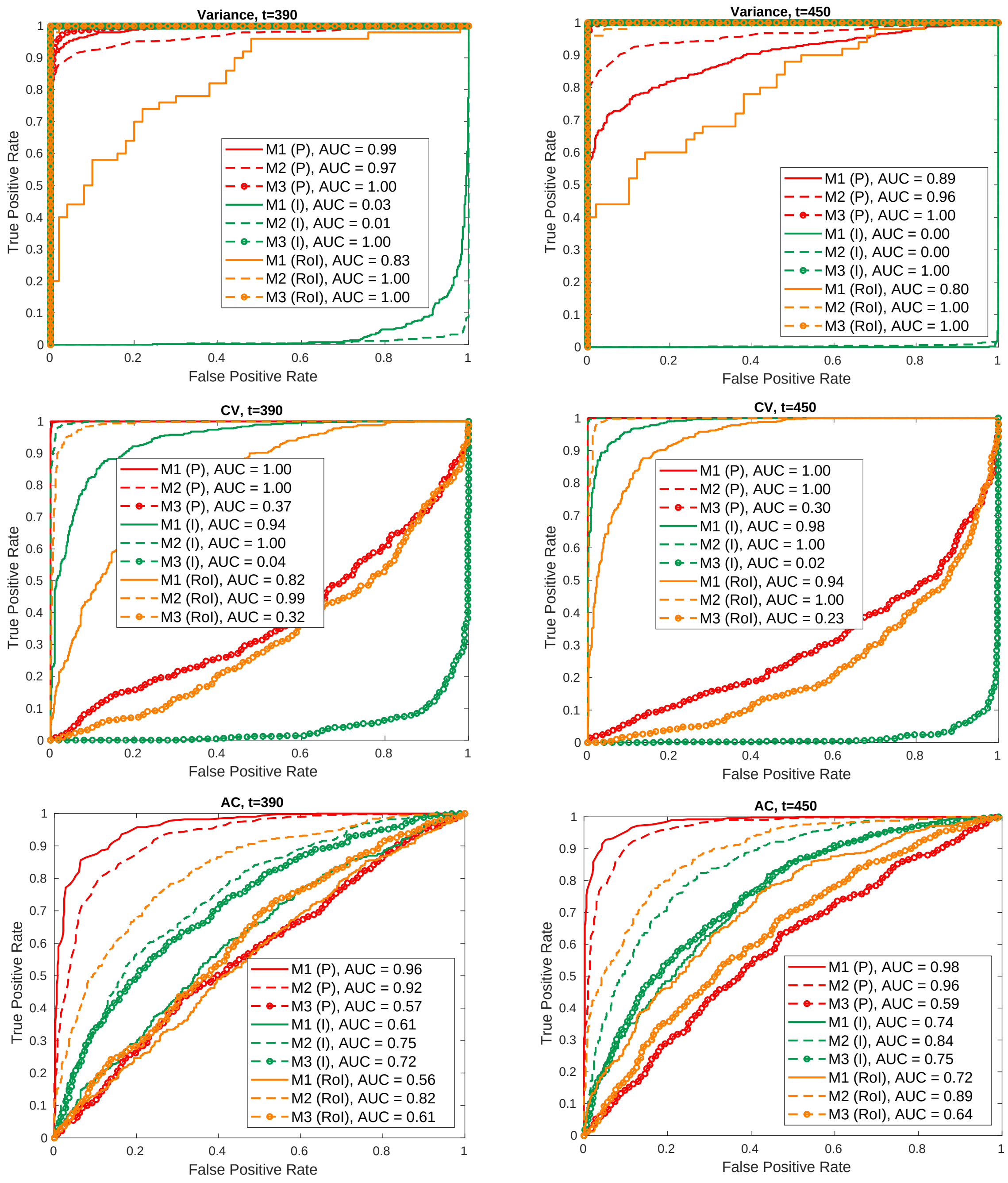

Fig. S10A. ROC curves for indicators V, CV and AC(1). Comparison of disease transitioning simulations: SIS with social distancing (solid lines); SIS with vaccination (dashed lines) and SIS disease emergence with increasing transmission (dotted lines) to the null model (Fixed endemic simulations). Each curve compares the null simulations to each model for different indicators and for each data type: prevalence (P, red lines), incidence (I, green lines) and rate of incidence (RoI, orange lines). ROC curves calculated for indicators: variance; coefficient of variation (CV) and lag-1 autocorrelation (AC). Kendall-tau scores measured over each data-type up to $t_1 = 390$ (pre disease transmission) are given in the left column and up to $t_2 = 450$ (post disease transmission) are given in the right column. For each ROC curve we measured the AUC score which is given in legend, which is an indication of how predictive each indicator is by its ability to distinguish between elimination simulations and the null model.

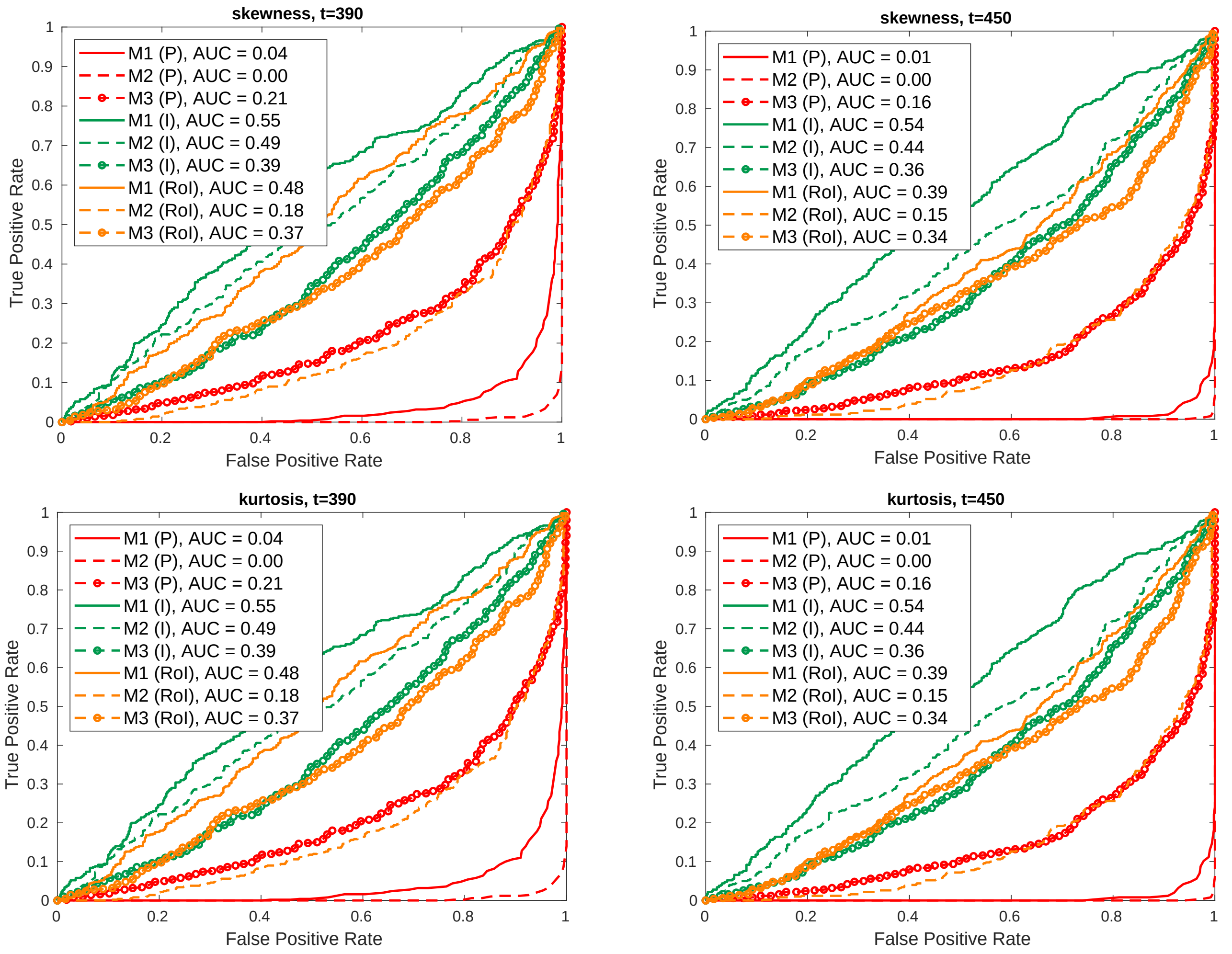

Fig. S10B. ROC curves for indicators Skewness and Kurtosis. Comparison of disease transitioning simulations: SIS with social distancing (solid lines); SIS with vaccination (dashed lines) and SIS disease emergence with increasing transmission (dotted lines) to the null model (Fixed endemic simulations). Each curve compares the null simulations to each model for different indicators and for each data-type: prevalence (P, red lines), incidence (I, green lines) and rate of incidence (RoI, orange lines). ROC curves calculated for indicators: variance; coefficient of variation (CV) and lag-1 autocorrelation (AC). Kendall-tau scores measured over each data-type up to $t_1 = 390$ (pre disease transmission) are given in the left column and up to $t_2 = 450$ (post disease transmission) are given in the right column. For each ROC curve we measured the AUC score which is given in legend, it is an indication of how predictive each indicator is by its ability to distinguish between elimination simulations and the null model.
