# Supplementary material for: Prospects for detecting early warning signals in discrete event sequence data: Application to epidemiological incidence data
Source: PLoS Comput Biol. 2020 Sep 22;16(9):e1007836. doi: 10.1371/journal.pcbi.1007836 (PMC7531856; doi:10.1371/journal.pcbi.1007836)
Supplement: S11 Fig — We compare the performance of 5 common statistical indicators for the SIS model: disease elimination with vaccination. For each ROC curve we measured the AUC which is an indication of how predictive each indicator is by its ability to distinguish between elimination simulations and the null model. The closer to 0.5 signifies the worst performance (random diagnosis). We evaluate the Kendall-tau score up to before the critical transition (t1 = 390) and after the critical transition (t2 = 450) which gives an indication if the EWS is increasing or decreasing. A score of 1 demonstrates that it is possible to identify all Ext simulations when compared to null simulations by its increasing trend (i.e. perfect sensitivity, true positive rate). A score of 0 means that there is zero sensitivity and instead the simulations are decreasing. (PDF) [file pcbi.1007836.s013.pdf]

AUC Results for SIS with vaccination model

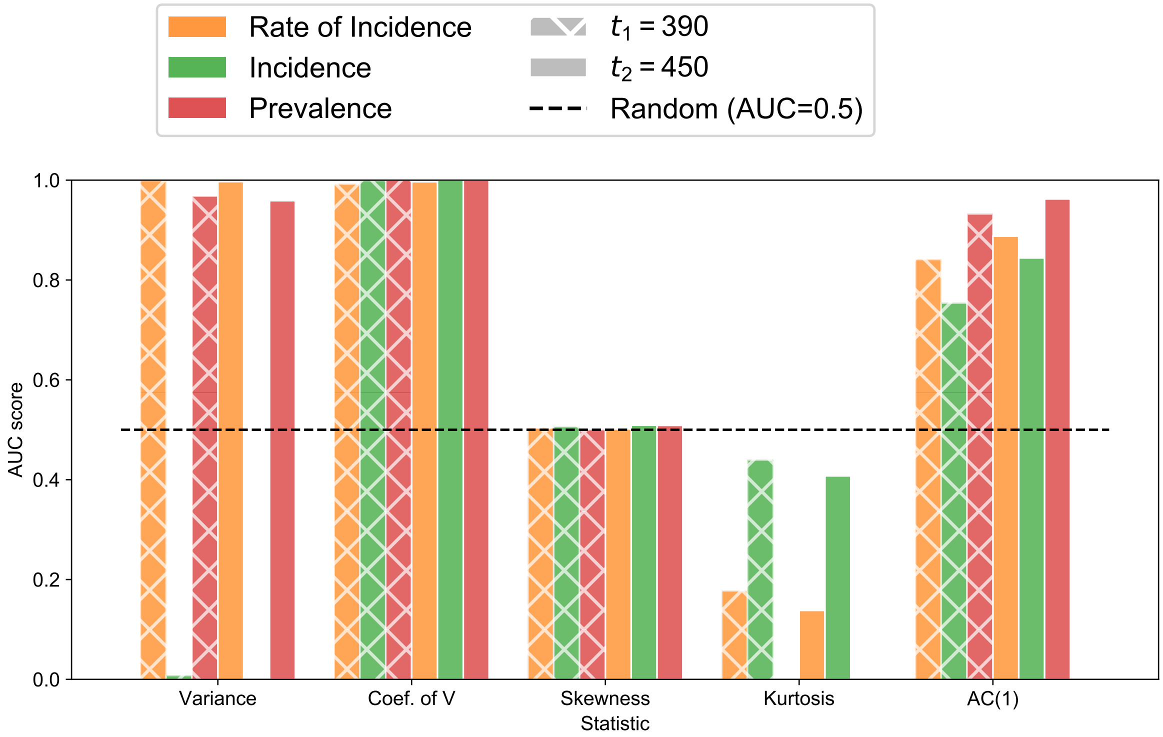

Fig. S11. AUC scores for different EWS (SIS with vaccination). We compare the performance of 5 common statistical indicators for the SIS model: disease elimination with vaccination. For each ROC curve we measured the AUC which is an indication of how predictive each indicator is by its ability to distinguish between elimination simulations and the null model. The closer to 0.5 signifies the worst performance (random diagnosis). We evaluate the Kendall-tau score up to before the critical transition ($t_1 = 390$) and after the critical transition ($t_2 = 450$) which gives an indication if the EWS is increasing or decreasing. A score of 1 demonstrates that it is possible to identify all Ext simulations when compared to null simulations by its increasing trend (i.e. perfect sensitivity, true positive rate). A score of 0 means that there is zero sensitivity and instead the simulations are decreasing.
